# Boundaries of the built environment: defining the significance of the material presence of spatial morphology in social life

**DOI:** 10.12688/openreseurope.16591.2

**Published:** 2024-06-10

**Authors:** Benjamin N. Vis

**Affiliations:** 1 Facultad de Arquitectura, Universidad Autónoma de Yucatán, Mérida, Yucatan, 97000, Mexico; 2 Faculté d’Architecture La Cambre Horta, Universite Libre de Bruxelles, Brussels, 1050, Belgium

**Keywords:** Inhabited built environments, ontology of conceptual boundaries, multidisciplinary theory, material affordance of social life, urban mapping

## Abstract

Settled societies inhabit environments shaped by building activity. Geographic data in social scientific and geographical research are generally composed of architectural and social categories derived from commonplace lived experience and societal knowledge, thus carrying socio-culturally specific meaning. The mundane pragmatism of such categories conflate spaces and buildings with their use and may obstruct effective comparison. Here I introduce a set of formally redescriptive ontological concepts for built environments that operates on the basis of how differentiation and subdivision constitute distinct occupiable spaces through boundaries. An ontology of the inhabited built environment arises from the application of these socio-spatial and material concepts called ‘Boundary Line Types’ (BLT). I present and photographically illustrate the definitions of the BLTs, which are conceived on a critical realist basis and rooted in a multidisciplinary body of theory concerning the development and inhabitation of built space. Considering inhabited built environments through BLTs foregrounds the emergent logic by which spaces are divided and connected, creating configurations of boundaries as material frames that afford everyday social life. Since BLTs offer transferrable empirical principles from which these material frames emerge, they also enable diachronic and cross-cultural comparative social research. My proposition to approach social scientific built environment research through constitutive material boundaries offers a comparative complement to commonplace and socio-culturally specific spatial categories that compose most geographic data, enabling formal thick redescriptions and the potential for quantitative spatial analysis.

## Introduction: boundaries as a vantage point

As human beings inhabit space, they section space both conceptually and physically. Depending on lifestyle, disposable materials, and technology, humans may choose to modify and eventually transform the physical characteristics of the spatial world to partition off sections in service of the purpose of inhabitation. In a habitable world, albeit not necessarily inherently hospitable, humans will use the experiential understanding of perceptible distinctions to direct and enhance their life-path within and traversing that world so as to survive and thrive by protecting themselves and their sustenance. Logically, the very first instances of construction that fully transforms the physical properties of the world will serve as shelter (cf. Appleton, 1975; Van der Laan, 1983). Such construction activity, modelled on previous experience of material properties (cf. constitutive phenomenology, Schütz, 1967), introduces a clearly perceptible physical contrast that generates a space where the vulnerability experienced in one’s surroundings is mitigated. Consequently, humans use their experience of differentiating between material properties to guide how they act upon the physical environment to create spaces dedicated to accommodating inhabitation, forming a spatial world that is habitable and intelligible. The spaces humans create exist empirically and ideationally. Importantly, spaces are shaped in constitutive relation to the social and physical environment within which they occur. Within local confines, social life in communities tends to create contiguous complex configurations of spaces that make up built environments over time.

This brief outline of the human condition of inhabiting the world highlights two essential principles. First, differentiation is situated as the embodied ( *sensu* Csordas, 1990) and emplaced ( *sensu* Howes, 2005) principle through which we come to know and understand the experiential world. Second, we make the world more habitable by transforming its physical properties (cf. Van der Laan, 1983), using our corporal and mental capacity in interaction with and acts on the environment. In this way, the process of inhabitation introduces physical subdivisions that differentiate spaces in relation to their surroundings: acts of construction leading to areas or spaces made to accommodate life that exert physical constraints. Understanding that built environments result from making the world habitable through the linked principles of ‘differentiation’ and ‘subdivision’ formalises descriptive parameters which can be used to meticulously conceptualise the role of built environment configurations in social inhabitation.

Here I will present an ontological set of socio-spatial conceptualisations of essential built environment elements. These concepts enable a redescription of the built environment based in fundamental, empirically observable, spatial-material information. The aim of accomplishing such rudimentary redescriptions is to advance the understanding of social life in constitutive relation to built environments, exclusively using as input data the spatial morphology (configuration, topology, composition, and shape) of the material environment that societies create for inhabitation. Focusing on the spatial morphology can avoid the cumbersome conflated categories in which we are customarily and culturally prone to perceive the built environment (see Lynch, 1984 ^1^). Customary categories of built environment elements tend to conflate the space and shape of buildings or areas with their specific function and meaning (such as church, house, or theatre). Meanwhile, the principles of differentiation and subdivision enforce us to think about how one space materially articulates its perceptible separation from the next. In other words, instead of thinking of discrete spaces or spatial entities as a *fait accompli*, they emerge from the way they distinguish themselves to their outside, which is an ongoing process in social life. This directs our focus towards the constitution of boundaries and what their material manifestation consists of ( Vis, 2013).

The ontological ( *i.e.* comprehensively redescriptive) definitions of types of boundary ( *Boundary Line Types* (BLT)) I will present, can be identified empirically in the spatial morphology of the inhabited built environment ( Vis, 2014a; Vis, 2018b). By adhering to transferable concepts to enable a formal redescription we can avoid the confusion of commonplace and cultural categories to describe built environment elements. Such formal redescriptions provide a foundation to conduct a comparative anthropology or sociology *of* places rather than *in* places (cf. Zimmermann, 2012). An empirical focus on spatial morphology enables near universal application based on the mapped, material record of places, which extends the comparative scope to informal settlements and into the ancient past, where the cultural specifics of life may be poorly known ( Harris & Smith, 2011; Vis, 2018a; cf. *space syntax* as developed by Hillier & Hanson, 1984; Hillier, 2007). The ability of any conceptual research approach to study long-term development is powerful. In the case of boundaries, long-term studies will likely deepen our understanding of the material entanglement of social life with the built environment (cf. Hodder’s, 2012 theory of entanglement), revealing how built space emerges from a continuous process of formation in which boundaries are being established, negotiated, modified, and transformed. The emergence of particular patterns of boundary types composing any phase of the development of built environments could thus be indicative of determinant cultural values, social utility, or ecological conditions.

I position the interpretive merit of the ontological typology of boundaries presented here as enabling studies into the significance of the material presence of spatial morphology in structuring social life and the subsequent development of places. Through the causal (restricting and enabling) powers of boundaries we can elucidate the interdependencies of the social, spatial, and material condition of humans and societies over time (cf. Fletcher, 2004; Hodder, 2012; Vis, 2009; Vis, 2018a: 50–57; Wallace, 2011). Fundamentally, the socio-spatial experience of the causal powers of boundaries in the built environment present themselves as the binary ‘inside-outside’. This binary highlights the paradox that exactly that which divides is also what connects. Boundaries determine the material properties of the division and connection between spaces. In everyday life, the material properties afford the restriction and enabling of relationships across the boundaries. In principle, all boundaries of the built environment can be crossed, albeit at times inevitable to disrupt their physical state doing so ( *e.g.* the Berlin wall). Boundaries are sites of mediation, facilitation, and contestation, enduring as a result of social acceptance and complacency and changing as a result of dissatisfaction, resistance, and developmental pressures, whether this applies to city walls or home improvement. By introducing the transferrable ontological definitions and illustrations to a cross-disciplinary audience, I aim to open new routes for interpretive and analytical comparative research on social life in built environments.

## Theoretical principles that ground boundary definitions

### Differentiation and subdivision

The ontological basis of the BLT definitions is formed by two rudimentary and linked principles of the human condition of inhabiting the world: differentiation and subdivision. The principle of differentiation is essentially nothing more than distinguishing between things. Differentiation depends on the acknowledgement that the extent of specific features or properties is limited. At this instance they become something else. Within the literature on limits, edges, borders, barriers, and boundaries the philosophical work of Deleuze and Guattari is regularly referred to. Yet, for the treatment of the material world, their dense geophilosophical language of space, including deterritorialisations, lines of flight, and smooth *versus* striated space ( Deleuze & Guattari, 1987), remains overly ideational and insufficiently concrete. Invested with the vigour of politics and power these ideas are evocative and thought-provoking, but in the context of the social experience of affordances in the built environment offer limited purchase. Instead, thinking on the materiality of objects and our embodiment in a physically manifest and transforming world offers a more productive philosophical vantage to approach the affordances of built space (see Woodward & Jones, 2005 for an ideational treatise of bordering and materiality). Subdivision is effectuated based on the experience and understanding gained through physical differentiation of the heterogeneous properties of the environment.

Subdividing transforms the world into distinct areas, producing a spatially heterogeneous environment composed of discretely delimited entities which are shaped to accommodate specific activities and relations. Focusing on the processes by which boundaries transform the physical environment into spaces allows us to deconstruct socio-culturally specific or commonplace and institutionalised discursive categories which are often found to compose mapped geographical data ( *e.g.* legal, commercial, civil, religious). Seeing how boundaries actively subdivide (and connect) spaces reveals a variety of affective and social responses (opportunities) to spatial distinctions that emplace built space within the constitutive experiential process of becoming.

This approach to boundaries as materially transforming our lifeworld finds more fruitful connection to cognate themes, such as dwelling or inhabiting ( *e.g.* Bollnow, 1961; Ingold, 2000; Ingold, 2008) and the structuring properties of the micro-geographies of everyday life ( *e.g.* Hägerstrand, 1975; Pred, 1977; Pred, 1981). Especially Pred’s ( 1984, 1986) inclusion of the transformation of ‘nature’ as constitutive part of the continuous and historically contingent processes of becoming of the subject, the social, and place has an immediate bearing on studying the built environment. I have called the physical transformation of space according to our experience of the world the materialisation of ‘human space’ ( Vis, 2009). In phenomenological terms, the process of material bounding to create discrete spaces essentially initiate with creating shelter; a rudimentary living space (cf. Appleton’s (1975) prospect-refuge theory). The delimitation of the structure that is realised contains relations to its surroundings and evokes strong affective responses as a shelter or dwelling. Paradoxically, the structure at once separates and connects its inside space from its outside space.

Producing a distinction between an inside and an outside is the most basic characteristic of architectonic space and in its simplest form emerges from placing two walls ^2^ in such a way that an area between them is formed ( Van der Laan, 1983). Van der Laan’s (1983) architectonic space is diametrically opposed to ‘experience-space’ by an internal relation, which forms a parallel with their external relation to ‘natural space’. Natural space is vertically oriented through the relation between earth’s surface and sky, while experience-space is horizontally oriented by human’s upright stature and movement. Adapted to the boundary thinking proposed here, the idea of experience-space is extended to comprehensively include the full phenomenological bi-implication of human-environment relations ( Ingold, 2000). In Van der Laan’s conceptualisation architectonic space introduces vertical intersections, while the boundaries between spaces can materially manifest themselves without vertical construction. ^3^ The material delimitation of discrete spaces reify the function of making the world habitable by uniting experience-space and architectonic space: experience-space ‘filling’ the inside space opposed to the outside ( Van der Laan, 1983). The relates to how Bollnow (1961) determines that lived-space becomes ‘total lived-space’ by creating the duality of inner and outer space through the building acts of erecting a house. According to Bollnow, humans realise their true essence by dwelling, which resembles Ingold’s (2008) notion that the nature of life is to inhabit the world.

The house or homestead, in whatever form, is the epitome of creating a space to live in. ^4^ It is no coincidence that Van der Laan (1983) resorts to the house as an exemplar elementary architectonic structure to explain his theory, nor that Bollnow (1961) esteems the house to the extent of ascribing ‘a certain sacred character’ to it. Furthermore, Bollnow argues, the ‘ *house* is the reference point from which he [humans] builds his spatial world,’ and: ‘although it [the house] is part of a larger whole, it remains the spatial centre of the life of the individual’ ( Bollnow, 1961: 32–33). More recently, Koch (2005) identifies the house and the family that resides in it as the core example when discussing socio-spatial systems as tied to the structural architectural system (representing material components, see Vis, 2009). Houses as places to live in are in their simplest form no more than a single room, as attested by Aztec ( Smith, 2008) and historical Maya ( *e.g.* Arana López, 2017: 72–73; Wauchope, 1938: 16–20) examples. Yet, they fully convey the materialisation of the principles of differentiation and spatial subdivision by physically transforming the environment into discrete inside and outside areas. Hillier and Hanson (1984) consider spatial subdivision in its simplest abstracted configuration to be the outline of a cell with relation to its outside. The means of entering and exiting that space (entranceway) may further specify the topological relationship to its outside.

### Boundaries of seclusion

Outlines, then, can be seen as the schematic, mapped representations of the boundaries as sites of difference ( Abbott, 1995) that shape the composition of the built environment ( Vis, 2013; Vis, 2014a; Vis, 2018a). As suggested by the word itself, a boundary imposes limits, which in built environments compose discretely bounded spaces. Exactly because bounded spaces do not exist in isolation but occur in an environment (thus maintaining a relation or relations to the outside), it is recognised that the significance of the material presence of spatial morphology in structuring social life and subsequent place development consists of how seclusion operates as a causal power. When outlines of spaces are mapped, they represent subdivisions that in reality are demarcated and characterised by the material properties encountered at the boundaries of each space. The seclusion (causal power) each boundary effectuates is always relative to the context in which it occurs and to the embodied and emplaced conditions and abilities of human beings encountering and experiencing it. Importantly, this makes us acutely aware that spatial configurations abstracted as outlines (cf. the empirical foundations of *space syntax*. See Hillier & Hanson, 1984; Hillier, 2007; Vis, 2016) can never fully capture the features and complexity of their material properties.

The broad socio-spatial significance of seclusion in social life is readily acknowledged. Consequently, one may argue that how (built) boundaries seclude should engage with a full understanding of the breadth of their ideational and emotive affects, as well as their empirical affects and affordances. Frequently, boundary research in the social sciences concerns socio-culturally specific themes (such as politics, religion, and economics), occurring in various guises from implicit social boundaries and categories in Van Gennep’s and Turner’s anthropology of rites of passage and symbolism (see Bell, 2009; Turner, 1969) to more explicit boundaries of organisation, territory and international borders ( *e.g.* Abbott, 1995; Jessop *et al.*, 2008; Jones, 2009; Lamont & Molnár, 2002). Indeed, the study of boundaries is at least as protean as its common usage as a term. So, it is important to be explicit about the delimitations of boundary research based on built environment maps and material records that I propose.

In my efforts to define transferrable BLTs the affective (emotive or sensory) properties and socio-cultural particularities of boundaries become subsumed in the rudimentary functioning of boundaries in social life and experience. Rudimentary social functioning implies a focus on the causal effects of encountering, introducing, adjusting, and crossing (or relating across) boundaries in everyday social life in built environments. Put differently, it focuses on how the seclusion of boundaries frame the interaction opportunities that structure society (cf. Vis, 2009: social positioning of spatialities) by formalising the properties of the interfaces of or connections between spatially distinct interaction opportunities. Empirically, the definitions must allow for the morphological, topological, and contextual identification of BLTs through which distinct patterns of interaction opportunities afforded in the inhabitation of the built environment become explicit. As such, the identification of BLTs generates a morphology of connectors in the built environment. It is anticipated that in BLTs empirical application and triangulation with other research (see Vis, 2018a: 45–66 on critical realism) the ontological redescription of built environments enabled by BLTs permits detailed, robust, and comparative understanding of the role of spatial morphology in social life, experience, and development, from which insights into socio-cultural particularities, functions, or values may emerge. In addition, the formal redescription or elements thereof may enrich questions on cognitive, emotive, or sensory responses to built space.

For a long time, non-human aspects or the materiality of space have stayed underdeveloped in the social sciences (excepting anthropology), as the nature of space poses a challenge to socially abstracted perspectives ( Delitz, 2009: 39–81; Delitz, unpublished work; Löw, 2008). This has been changing since the wider adoption and application of the material-semiotic method of Callon’s and Latour’s Actor-Network Theory, which places human beings and objects symmetrically in network relations. Anthropology, however, has retained an enduring and diverse relationship with the material or physical aspects of the world (see Miller, 1998; Miller, 2005). ^5^ Lawrence and Low (1990) synthesised influential anthropological thinking on the relation between the social and the built environment or built form. In the Anglo-American tradition the work of Rapoport (especially 1982) has been influential for maintaining anthropology’s interest in architecture. ‘Humans tend to segment or partition an undifferentiated, continuous environment into bounded space. The environment can be partitioned conceptually through the habitual use of specific activity areas either inside or outside dwellings; environment also can be partitioned physically by means of walls [...]’ opens a paper by anthropologist Kent (1991: 438) on cultural variation in domestic spatial organisation. In 1996 Pellow dedicated a volume to refocus anthropological investigation specifically on boundaries, containing notable contributions which actively refer to boundaries’ physical side ( *e.g.* Lawrence, 1996; Rotenberg, 1996), devoting much attention to planning and housing.

Notwithstanding the utility of network thinking, the logic supporting BLTs bears stronger resemblance to Hodder’s (2012) asymmetrical human-thing relations in entanglement. However, the concrete foundation of their definitions ( Vis, 2018a) combines constitutive phenomenology ( Schütz, 1967) with Gibsonian ( Gibson, 1979) affordance to precisely articulate their material character using archaeological adaptations of critical realist philosophy ( Wallace, 2011). The socio-spatial causal powers of boundaries are recognised in topological relations to their environment through empirical iterative abstraction ( Sayer, 1981; Sayer, 2000; Yeung, 1997). Meanwhile, the emergent nature of boundaries’ material character in constantly developing environments made for inhabitation produce a perspective on the built environment that recalls Pred’s ‘transformation of nature’ and Bourdieu’s *habitus* ( Bourdieu, 1977; Pred, 1977; Pred, 1984; Pred, 1986; Vis, 2018a; cf. Wallace, 2011).

The archaeological basis of the ontological typology of boundaries ensures their applicability to mapped representations of built environments and their material record. On the one hand this empirically maximises comparative potential without cultural preconceptions about architectural form and building usage. On the other, this enables integration with other formal spatial analytical methods as well as triangulation with additional or alternative socio-cultural, cognitive, or emotive data.

## Formulating empirically recognisable boundary concepts

### Specifying the ontological nature of boundary line types

The following section summarises a few specific requirements and stipulations that the ontological boundary line type definitions should adhere to or simply respect in their application. Seeing the built environment as inhabited means human beings are emplaced in built space. Thus, we are always enveloped or framed by the boundaries that bound the space we find ourselves in. This is important for how we conceptualise boundaries, because it determines that boundaries we recognise always manifest from the inside looking out or from the outside looking in. If the affordance of a boundary to be crossed is realised, *i.e.* a human being crosses the boundary, this relationship is inverted. Put differently, each boundary or even each line has two sides and therefore the definition of any boundary line type needs to be considered topologically and contextually from both sides. Moreover, given that multiple spaces may emerge from built boundaries on either side, no outline that circumscribes a unit of built space can ever be defined by only a single boundary line type. A comprehensively defined boundary is contextually dependent on exactly what emerges from the spatial morphology present on both sides. In other words, each subdividing line representing a boundary will automatically require a double definition; one resulting from the seclusion occurring on the outside and one defining the spatial unit on the inside. Considering that one can imagine material boundaries subsuming or uniting a whole subset of boundaries ( *e.g.,* city walls, walled compounds), any subdividing line might require additional definition to take into account the mereology ^6^ of spatial morphology.

As indicated previously, the way the causal power of seclusion operates depends on the material properties of each boundary. It also follows that if certain material properties or compositions are absent from a given built environment, certain BLTs or BLT combinations need not occur at all. Naturally, a comprehensive consideration of material properties could account for tremendous levels of detail, including architectural textures, style, form, tradition, and construction ( *e.g.* Kropf, 1996). However, because of my comparative aims the relevance of detailed material properties will be curtailed by the rudimentary social functioning that enables comparative application. It should be noted that a focus on rudimentary social functioning of boundaries has a deliberate limiting effect, by excluding all kinds of sensory and emotive affects brought on by material properties such as colour, decorations, depictions, and material use, to name a few. In other words, the boundary line type definitions, although explicitly material in nature, do not purport to support an all-encompassing redescription of the material construction of built environments. The possibility of adjusting or adapting my boundary conceptualisation to accommodate additional and alternative empirical information is not precluded. In addition, for purely practical and comparative benefits I have limited myself to a ‘close-to-ground level’ experience of spatial morphology, which also tends to correspond well with the information conveyed by conventional ground level (building footprint) town plans. These limitations, while not catering to all, also permit that informed practitioners could apply boundary line type definitions to most built environment maps without much background research.

At a level of rudimentary social functioning, it is evidently essential to distinguish how the material properties of boundaries permit them to be crossed, and thus to change one’s socio-spatial position and associated interaction opportunities. The paramount assumption is that, because the built environment results from human construction, in principle any boundary is crossable and, therefore, open, accessible or permeable. Material properties of ‘open’ boundaries can be seen as passive (permeable or intermittent) in that they readily allow traversing. However, the physical properties of many architectural constructions counter this assumption, since boundaries may literally have to be broken in order for a crossing or direct interaction to take place. Thus, the seclusion afforded by the structural integrity of walls (or elaborate fences) is pragmatically assumed to be impermeable. Such boundaries form barriers to regular crossings. Undisruptive interaction between the sides concerned is not possible. This renders the material property of impermeability of boundaries of great importance in their definitions. It would make a logical extension to consider material properties of boundaries that mitigate impermeability: that readily permit forming and enacting relationships across the boundary without any party actually crossing it. This could be related to various senses, such as vision and hearing, which invest windows, hedges, fences, or construction material with socio-spatial significance. Determining to what extent material properties are permeable I deem currently impractical and an impediment to the optimal comparative application of boundary line type definitions. ^7^

Giving the operation of seclusion ontological primacy makes that the first boundary line type definition will determine the ability to close off (make impermeable) a space from undisruptive interaction from the outside, emphasising an internal assertion of seclusion. That notion of closing off from the inside towards the outside immediately derives from the rudiments of architectonic space as shelter or a simple cell (cf. Appleton, 1975; Hillier & Hanson, 1984; Van der Laan, 1983) and the occupation of a constructed space by a specific socio-spatial system (cf. Koch, 2005). A material articulation of extracting a discrete space from its surroundings also exerts a mundane spatial power relation, because the same space cannot be occupied twice ( Hägerstrand, 1975; Pred, 1977; Pred, 1981: neither by construction nor people). Thus, impermeable seclusions dominate the space they occupy and may cause spatial morphological associations of dependence with surrounding boundaries. There is an intrinsic interest in entranceways, because they create orientations in how dominant spaces are connected to their surroundings by facilitating solicited or voluntary interaction. Doors and gates as entranceways have material properties that afford closeability, effectively rendering a stretch of boundary reversibly (im)permeable ( *i.e.* the temporary manipulations of opening, closing, and locking a door, cf. Latour’s (1992) wall holes and their reversible state resulting from door hinges). ^8^

The boundary line types will distinguish a dominant and a solid dominant, in which the latter refers to a single cell configuration ( Hillier & Hanson, 1984) or the single room house. When considering architectural information in typical town plans buildings or open spaces are merely outlined; disregarding internal organisation or design. The prefix ‘solid’ alerts us that there is no further differentiation inside, which on the flipside permits the mereological possibility of a spatial hierarchy where a closable boundary can enclose a subset of boundaries.

From the premise of an inhabited built environment follows that the spaces that emerge from boundaries must be occupiable to a degree of permanence by a specific socio-spatial system pertaining an activity or set of interactions (see Vis, 2009; cf. Koch, 2005). This precludes the occurrence of spaces that cannot be occupied, even though such spaces do exist within intensively developed built environments ( *e.g.* electrical substations, water towers, steep slopes or challenging rocky outcrops, bodies of water). Socio-spatially speaking, such boundaries of unoccupiability generate ‘negative spaces’ in the social complex, which means they are void of occupation, but not necessarily of significance. Therefore, the inclusion of negative boundary line types will ensure that the spatial morphology of built environments can be defined contiguously and comprehensively, thus upholding their ontological claim.

Ultimately, it is essential that each boundary line type definition will be empirically identifiable, be that on town plans or walking around town. Yet, as a consequence of the above requirements and stipulations, a single boundary line type identification is never capable of fully defining and thus redescribing the spatial morphology in terms of rudimentary social functioning. While topographical data represent an abstraction of material boundaries based solely in observation of their physical presence, applying BLTs to these boundaries will de- and reconstruct them according to empirically recognisable conceptualisations that are no less abstract. Information selection and abstraction is exactly what renders mappings useful (see MacEachren, 2004; Monmonier, 1996; Wood, 1992) and similarly the conceptual ontology that emerges from the identifications of BLTs is not designed to express the entire concrete truth (cf. Sayer, 1981). We recognise that boundaries should always be considered from two sides and that there may be mereological relationships and topological specifications that result from their material and morphological properties. Therefore, it becomes an expectation that the significance of spatial morphology to the inhabitation of built environments is only revealed through combinations of BLTs operating along the same boundary. The fact that boundaries do not become defined by a single act of defining also stresses their emergent logic and complexity.

The BLT definitions are presented accompanied by photographs of street scenes in aid of improving understanding of the formal analytical language used. These photographs allow me to demonstrate their general applicability unencumbered by the particularities associated with using topographical data which require knowledge of their original purpose and construction (see Vis, 2014b).

### Boundary Line Type (BLT) definitions

This section defines and discusses the thirteen BLTs (numbered and named) that I currently recognise. The initial order is determined by departing from the simplest occurrence of seclusion generating a discrete space: the solid dominant (cf. Hillier & Hanson’s (1984) single cell configuration). As such the numbering is generally representative of the iterative abstraction process (cf. Sayer, 1981; Sayer, 2000; Pratt, 1995; Yeung, 1997) through which they have been identified and do not express any further meaning. The BLTs have also been named in active voice to express their role in the inhabitation of the built environment. In line with iterative abstraction, their ontological conceptualisation is deemed practically adequate by having been tested through several instances of application ( Vis, 2014b; Vis, 2018a), but remains forever open to revision and refutation depending on empirical findings. Note that no BLT need to occur in any given built environment and that only through their relations do they describe boundaries that compose the spatial morphology of inhabited built environments. Since the language used should avoid being socio-culturally prescriptive, the comprehensive definitions (first presented in Vis, 2018a: 141–158) may come across formulaic, complex, and descriptive. To aid understanding and to promote their use, in this paper I reproduce their abridged definitions in Table 1 (adapted from Vis, 2018a: supplement), followed by a discussion based on a set of annotated photographic illustrations.

**Table 1.** Abridged BLT definitions.

Boundary Line Type (BLT)	Empirically identifiable principle	Social relation to interaction opportunities	Indicative contemporary urban example
**1** **Closing boundaries**	Operates on the basis of seclusion of a continuous spatial arrangement from the surrounding configuration with the material property that the boundary can be closed off towards its outside, thus making it a dominant. It is also a solid ( *i.e.* no internal arrangement of outlines)	Interaction opportunities are quite stringently internalised as distinct from the outside, though there is a mutual (in)direct orientation between the solid dominant and the surrounding configuration	These boundaries typically circumscribe buildings of any sort or size
**2** **Facing boundaries**	Operates on the principle of the orientation for soliciting interaction from the surrounding configuration	Is the site of solicitation of interaction with a dominant	These boundaries represent the doorways or entrance ways into a building
**3** **Associative boundaries**	Operates on the basis of dependence on a single dominant that it is directly associated with and, in a conjunction including possible other (in)directly associated boundaries, with which it forms an adjoining configurative complex	Interaction opportunities are mediated between the openness of the surrounding configuration and the related dominant	These boundaries are typically associated with gardens or any plots and surfaces belonging to a specific building
**4** **Extended facing boundaries**	Operates on the principle of orientation in an uninterrupted connection to a facing boundary by dependence on any boundary associated with a dominant	Is the site of indirect solicitation of interaction with a dominant, proceeding is no necessity	These boundaries are typically associated with garden gates or courtyard entrances, etc.
**5** **Directing boundaries**	Operates on the basis that it directs interaction along opportunities for further boundary crossings in parallels	Interaction opportunities are directed along the boundary crossings that constitute its sides, connecting all sorts of bounded spaces	These boundaries are associated with the street network, access and pathways
**6** **Disclosing boundaries**	Operates on the basis of guiding interaction towards opportunities for further boundary crossings in multiple directions rather than a single particular direction with necessary (in)direct connections to solid dominants	Interaction opportunities are freely organised, yet directed in multiple directions which in several cases will eventually lead to soliciting interaction with solid dominants	These boundaries are associated with square-like spaces in well integrated urban situations with several associated buildings
**7** **Enclosing boundaries**	Operates on the basis of seclusion from the surrounding configuration with the material property that the boundary can be closed off towards its outside, making it a dominant while containing solid dominants	Interaction opportunities are restricted by solicitation between the openness of the integration within the boundary configuration and the configuration with solid dominants that it circumscribes	These boundaries are typically associated with city walls and gated communities
**8** **Mutual boundaries**	Operates on the principle that it is simultaneously associated with, or encompassing, a distinct subset of several solid dominants with which it forms a configurative complex	Interaction opportunities are indirectly directed to several solid dominants and mediated between the openness of thoroughfare	These boundaries are associated with a specific group of buildings without any preference as to which it provides access such as shared porches, cul-de-sacs and communal space in gated communities
**9** **Opening boundaries**	Operates on the principle that it creates open, accessible connections towards its outside, while being an integrated part of the configuration	Interaction opportunities are freely organised, with no prerequisites for boundary contexts and the possibility of thoroughfare	These boundaries can be described as park-like spaces, *e.g.* garden plots, urban fallow, parking surfaces
**10** **Neutral boundaries**	Operates on the principle of neutrality, which results from ambiguity and the absence of singular associations, and can occur in virtually any context	Due to the absence of an unambiguous relation to a residing socio-spatial system, crossing the boundary creates no difference from the surrounding non-dominant configuration	These boundaries tend to be the left over areas in less optimally used built environment configurations and also some delimited functional areas connected to streets ( *e.g.* electricity supply)
**11** **Man-made boundaries of unoccupiability**	Operates on the basis of negativity, can occur in most contexts	Negativity means there is no residing socio-spatial system, in this case because an area cannot (structurally) be occupied by human beings	Structures that create an unoccupiable surface area, such as ponds, canals, architectural talus, narrow gaps, etc.
**12** **Not man-made boundaries of unoccupiability**	Operates on the basis of negativity, can occur in most contexts	Negativity means there is no residing socio-spatial system, in this case because an area cannot (structurally) be occupied by human beings	Steep slopes, natural bodies of water, etc., which are contained in the built environment
**13** **Not man-made negative boundaries**	Operates on the basis of negativity, can occur in most contexts	Negativity means there is no residing socio-spatial system, in this case because it marks the end of the built environment	'Nature': wild or not fully cultivated areas, or in some cases extensive areas of (urban) fallow or agriculture
**V** **Virtual boundaries**	Sites of distinction afforded by extant physical distinctions, human beings would have understood and/or experienced to be a crossing from subdivision into subdivision without clear material markers imposed onto the surface	Can in principle be part of any BLT that is not closable or negatively defined	Locations of crossings from space to space are in principle unimpeded and predominantly unmarked, such as openings in dry stone walls circumscribing fields, or a cul-de-sac connecting to a street with similar surface

The ontological formulations of the BLTs, based on how boundaries operate, ensure they are useful in comparative application along the lines of Kropf’s ( 2009, paraphrased) insistence on a consistent, coherent and comprehensive set of definitions, which are general enough to be used comparatively in all thinkable contexts, yet specific enough to be identified as analytical units in the empirical reality of datasets. When applied, the BLTs will still resemble the spatial entities that socio-culturally specific or commonplace categories in geographical data ( *e.g.* house, street, garden, *etc*. as indicated in the final column of Table 1) recognise, but these entities will have been redescribed. The emergent definitions of each boundary composing the spatial morphology of the built environment are proposed as a detailed spatially and materially explicit canvas for social and cultural studies.

## Illustrating how Boundary Line Types occur in built environments

### Closing & facing boundaries (1, 2)

**Figure 1.**
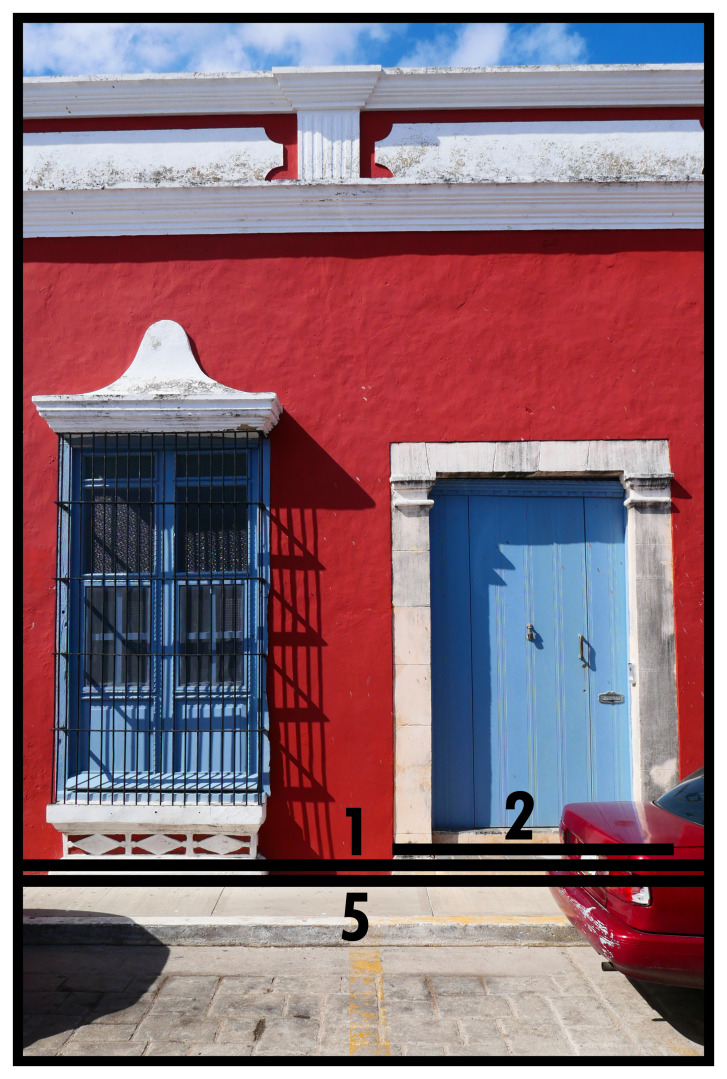
Boundary Line Types 1 and 2 in context (Type 5).

Figure 1 depicts a common manifestation of the simplest BLT configuration. The emplaced photographic perspective means we only see the façade of a built volume (solid dominant) which would be fully circumscribed by an outline identifiable as Type 1. The façade in this instance is part of a series of joint adjacent façades (terraced housing), where the internal divisions (empirically perceptible, but not directly visible) separate built volumes to accommodate separate socio-spatial systems. The doorway materially specifies its relationship to its outside and is identified as Type 2. The construction of Type 2s introduces material properties that, at will, allow traversing into dominants or close them off stringently. Type 2s depend on the solid dominant created by Type 1 or the dominant created by Type 7 and can occur multiple times, potentially generating multiple orientations towards the outside ( *e.g.* back doors, multiple entrances in larger buildings such as offices, or multiple city gates). In this instance, the Type 2 directly connects to the street space, identified as Type 5, which creates an instantaneous exposure to social interaction from all origins and immediate possibilities to pursue interactions with other socio-spatial systems thus connected. To avoid dominants turning into a negative boundary, thus extracting the space from social interaction, it is a prerequisite to have at least one Type 2. Type 2s channel interactions sought with the socio-spatial system residing in a Type 1. The crucial aspect of identifying Type 2s is that they facilitate crossing into the dominant and can be closed, so they are not to be confused with architectural frontage.

It is prudent to note that when BLTs are used without further documentation, an apartment building where multiple housing units use a common entrance is not immediately distinguishable from single (large) houses or other major buildings with one or multiple functions. Buildings with multiple layers (high-rise) exacerbate this phenomenon. In the interest of broadening comparability across time and cultures this ambiguity in architectural housing units is currently left unresolved ( *i.e.* a built volume represents all socio-spatial functions accommodated within combined). This leaves flexibility for resolving this in empirical practice dependent on research purpose. Similarly, it would be possible to document entrances in detail, permitting distinctions in Type 2s according to an architectural typology based on stylistic, functional, technical, symbolic, or economic aspects of construction (cf. mapping architectural interfaces in Dovey *et al.*, 2017). Generally, increasing levels of detail diminishes comparative potential.

### Associative & extended facing boundaries (3, 4)

**Figure 2.**
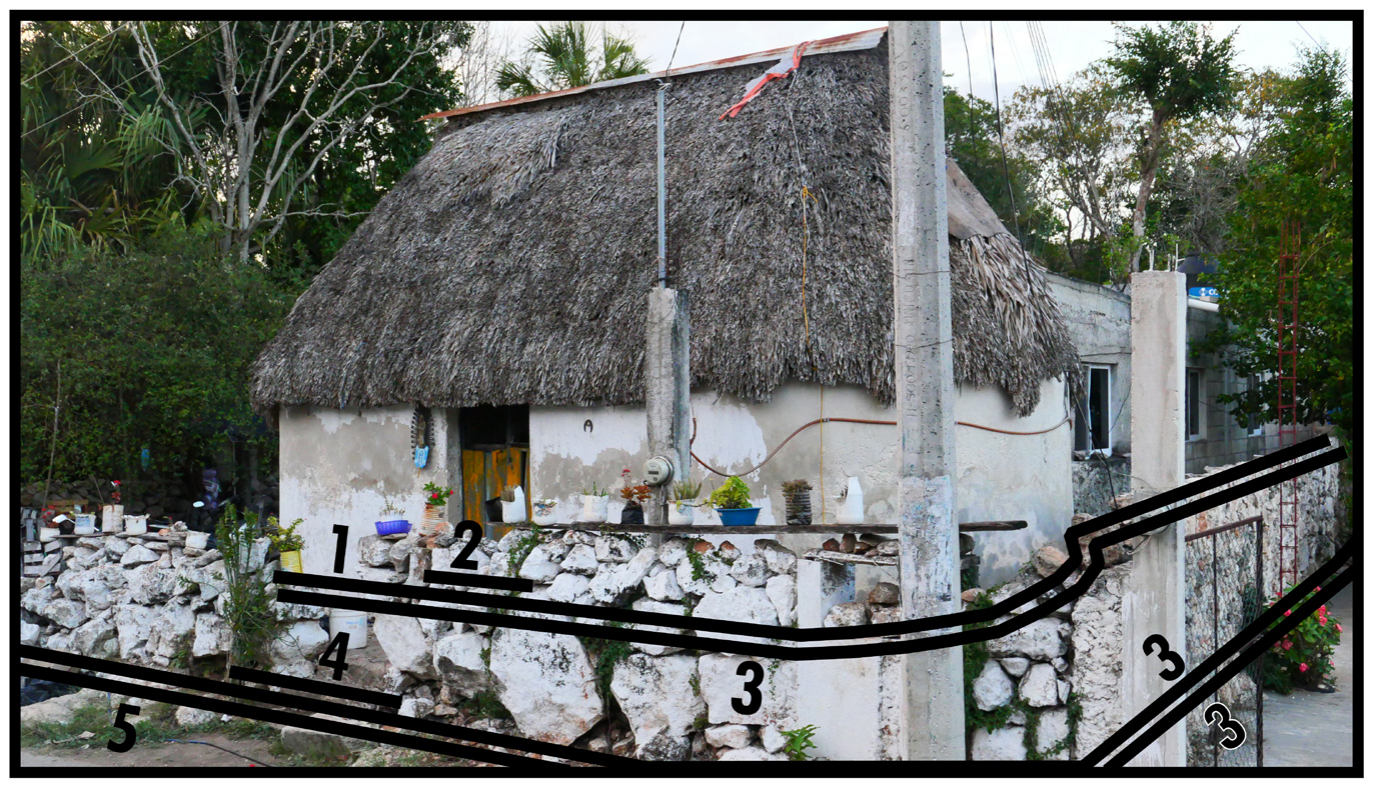
Boundary Line Types 1, 2, 3, and 4 in context. The additional Type 3 on the right belongs to a neighbouring configurative complex.

**Figure 3.**
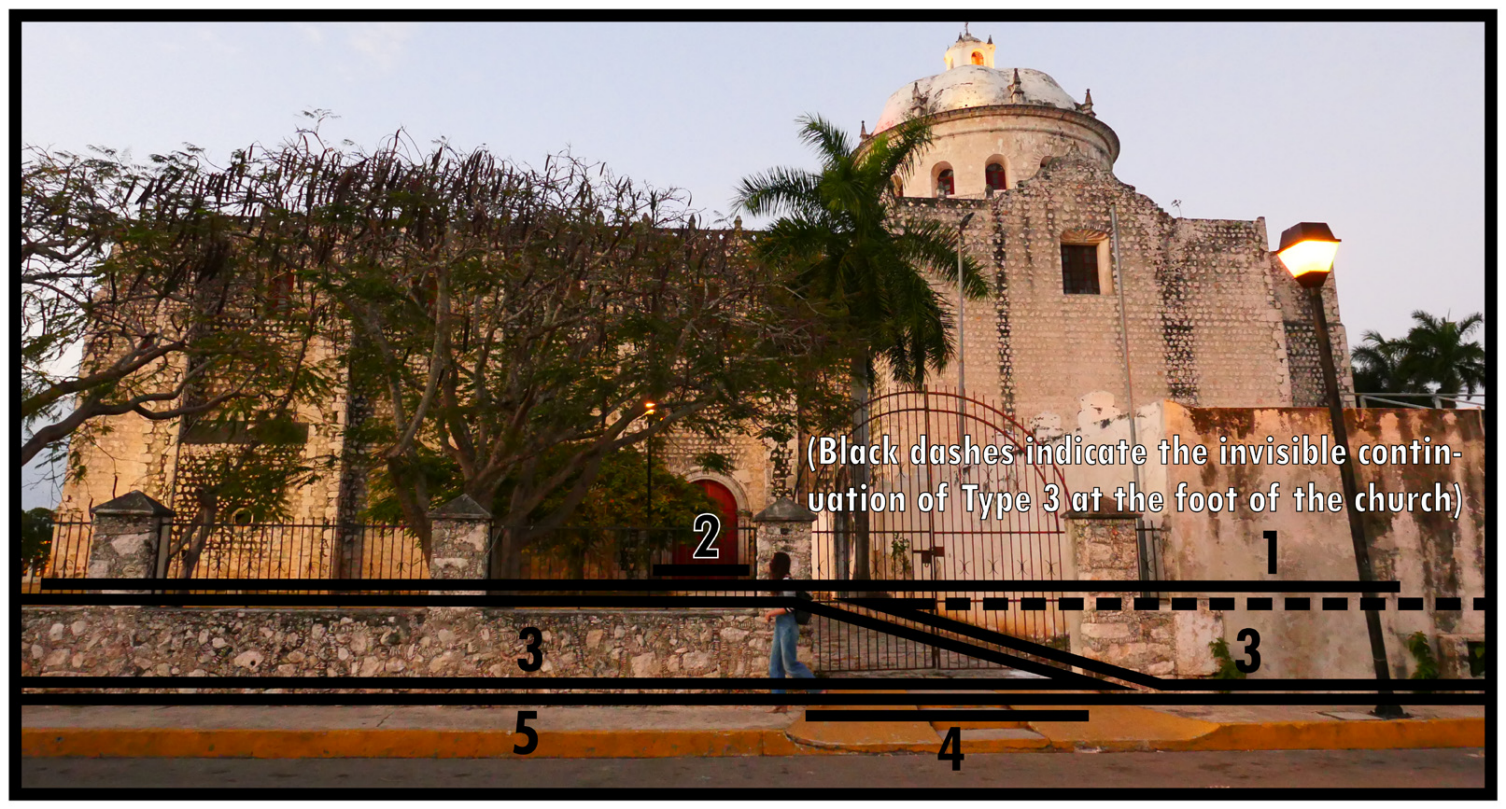
Two Boundary Line Type 3s in succession in context. Note the Type 3 on the right shows material evidence of impermeability.

Figure 2 expands our perspective to situate a solid dominant in a surrounding space which is directly associated with it: a building in its plot, which is a pervasive configurative complex in most contexts. A Type 3 is thus identified on the basis of its association with a dominant as a dependant. The configurative complex resulting from dependence on a dominant can be extended with multiple Type 3s and could potentially involve a Type 8 in addition. Type 3 boundaries are assumed to be open to crossings (thus materially permeable). With direct evidence for material construction that fully secludes the space, Type 3s could be regarded as extending the dominant they depend on (see Figure 3). Type 3s mediate the relationship between socio-spatial systems residing in dominants and interactions emerging from the surrounding inhabited built environment. The most common manifestation of such mediation is the (front) garden. Their protean morphology can cause ambiguity in identification, as spatial-materially it may not be clear how plots are (legally or practically) separated or when an outside space belongs to a dominant. In research it may be necessary to decide if functional outbuildings are to be subsumed in Type 3 identifications or documented as subsidiary structures.

The occurrence of Type 4s depends on Types 3 or 8. While it is similar to Type 2, Type 4s emphasise places along those boundaries that are easily crossed or constructed with the specific intention to be crossed there instead of the possibility of closing off the boundary stringently. When, as applies to the left-hand Type 3 in Figure 3, the closure is materially strong and preventative to unencumbered crossing, sensorily the closing of the space associated with the church is far from complete or inhibitive of interaction. Generally the relation to its outside is conducive to situations that ultimately permit interaction, generating a sense of mediated mutual orientation between the dominant and the surrounding configuration. Since BLTs refer to outlines of essential land-use divisions disregarding internal spatial arrangements, crossing a Type 4 should permit access to one or more Type 2s along one or more Type 1s without necessitating further boundary crossings. An extended facing boundary (entrance) along a mutual boundary (Type 8) followed by an extended facing boundary along an associative boundary (Type 3) is an exception.

Bear in mind that for an elaborate configurative complex consisting of multiple associated boundaries enveloping a dominant, the dominant can only connect to its surrounding configuration by means of a succession of a Type 2 and a Type 4 which on at least one *topological side* connects to the outside of that configurative complex. A topological side refers to a section of boundary along which the same BLT identifications operate, as determined from the emergent spatial subdivision on the outside. Regardless of geometry, boundary seclusions can have distinct topological sides determined by each unique instance of collocated BLT identifications. In Figure 3, for the right-hand Type 3 geometric and topological sides coincide according to the Type 3-5, Type 3-3, and Type 3-1 combinations annotated in the photograph. In Figure 2 the whole accreted built volume forms one topological side with the Type 3 that envelops it along the outside. Extended facing boundaries can consist of everything from simple gaps in vegetation or fencing to elaborate gates. Therefore Type 4s can be underdefined and informal, collocating with entire topological sides of the Type 3 or 8 in play.

### Directing boundaries (5) and virtual boundaries (V)

**Figure 4.**
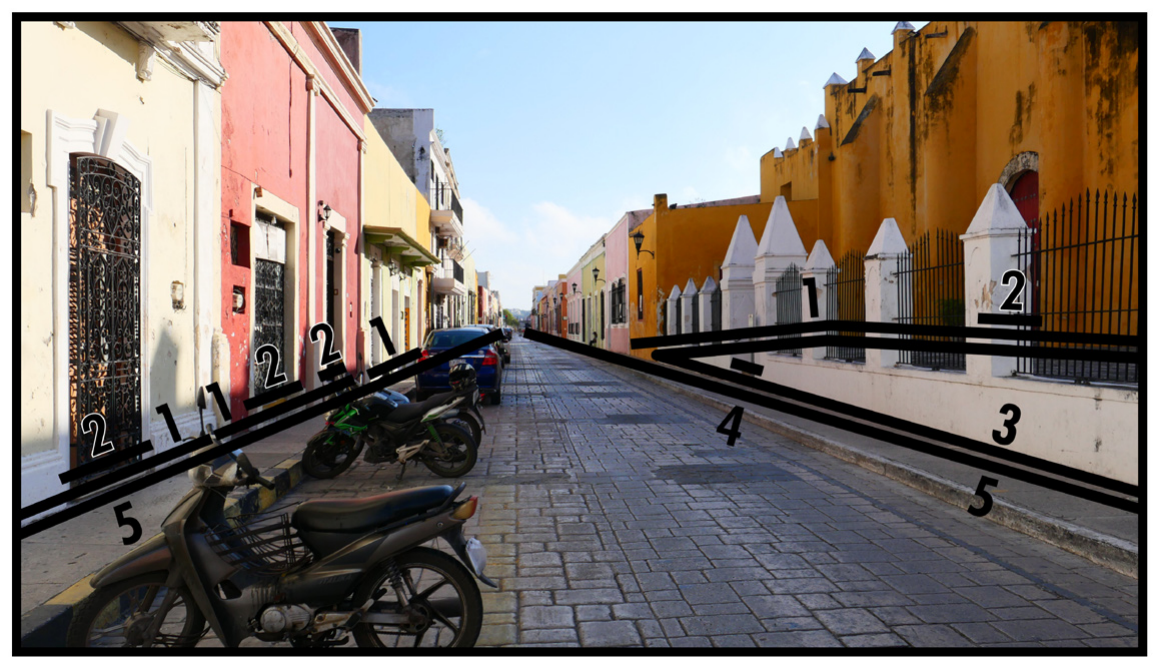
Boundary Line Type 5 in context.

As Figure 1– Figure 3 have shown, Type 5 pertains to the spaces that function as streets. In Figure 4 we appreciate a typical manifestation of a Type 5 in an area densely packed with spatial subdivisions. Here the Type 5 is flanked by architectural constructions, although usually Type 5s are involved in connecting all different kinds of BLTs in any built environment. Thanks to its flexible connections, Type 5s direct towards and away from any interaction opportunities at will. While street networks are recognised to be an essential part of urban built environment analyses ( *e.g.* space syntax ( Hillier, 2007) and urban morphology ( Conzen, 1960)) the BLT definition favours comparative flexibility which serves less geometrically structured built environments or networks of flows arising from different or informal configurations of space. (In practice the parallelism of Type 5s can also include short stretches of roughly mirrored divergence, maintaining an equal relationship of boundaries on both sides.)

**Figure 5.**
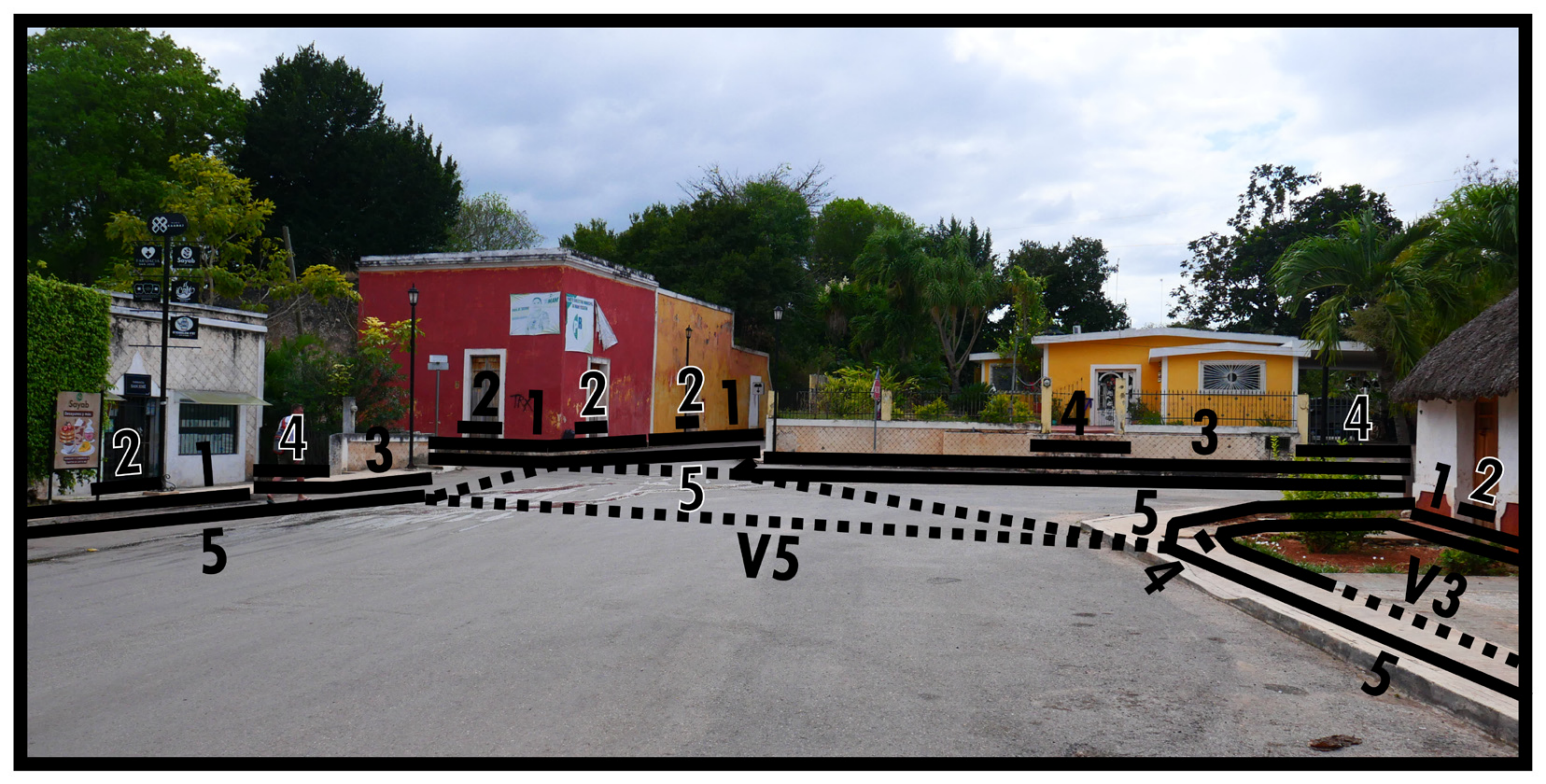
Virtual boundaries (dotted lines) in context.

Figure 5 shows that the parallelism of directing boundaries (Type 5) causes ambiguity when multiple parallels meet. At street crossings there are generally no significant material changes to space to perceive discrete separation and the same socio-spatial functioning persists, but interactionally the spatial-material context presents us with a choice of spaces (directions) to cross into. This kind of spatial subdivision, suggested by and experienced through spatial-material context, is more common than it might appear, for example should a street end by crossing into a different BLT identification. On the right-hand side of Figure 5 we see a virtual Type 3 delimiting the barely discernible difference between street and paved garden space. In fact, the Type 4 indicating the intended entranceway onto the plot is materially only emphasised by the lack of paving on either side of the path. The other garden spaces in this image are much more elaborately separated, indicative of a functional difference in how the Type 1s relate to the Type 5 in the foreground. Virtual boundaries are thus a virtual extension of an empirically differentiated boundary, without requiring actual material differentiation along its course. Such material informality in itself can indicate nuanced differences in socio-spatial functioning.

### Disclosing, enclosing & mutual boundaries (6, 7, 8)

**Figure 6.**
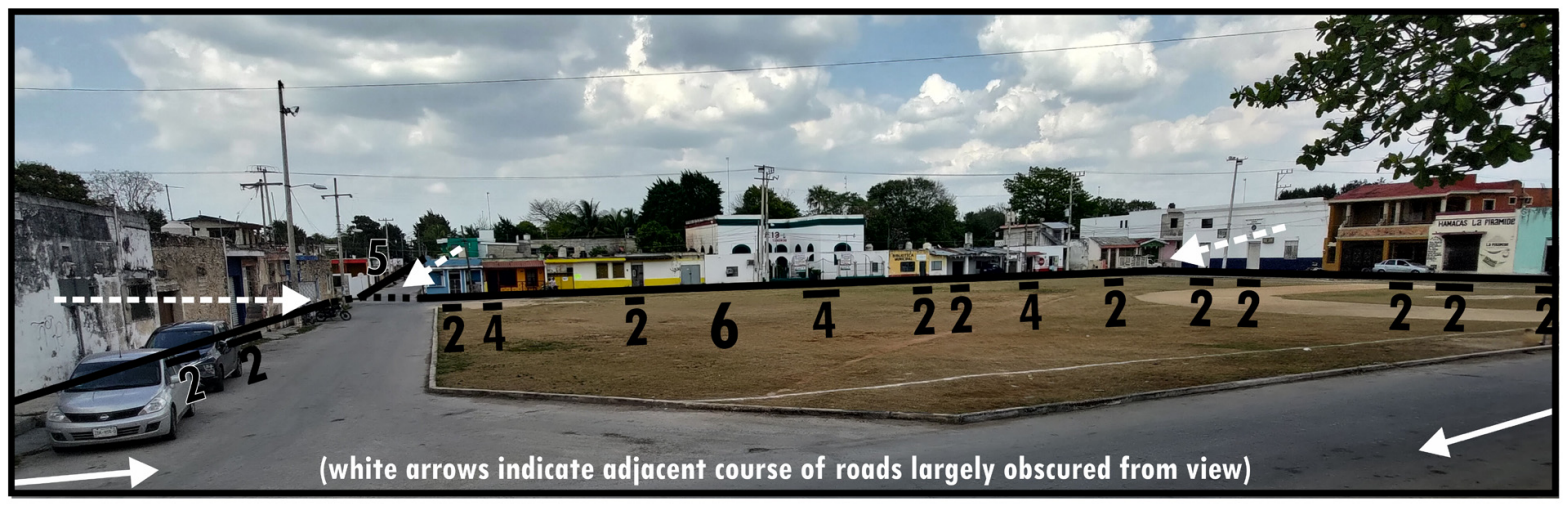
Boundary Line Type 6 in context (dotted lines indicate virtual boundaries).

The principle of disclosing boundaries (Type 6) is that they connect interaction directly or indirectly ( *via* Type 3 or 8s) to several dominants in multiple directions, generating a sense of local centrality. Because Type 6s create readily traversable spaces that are oriented to facilitate high integration with the surrounding built environment configuration, it avoids bringing these dominants together into a dependent configurative complex by social association. In other words, Type 6s disclose several dominants to public interaction, which we most commonly encounter as squares or plazas (see Figure 6). The practical dominance of motorised mobility oftentimes requires channelling of that mobility along pathways that materially continue Type 5s. This means, following a perspective geared towards transport, that Figure 6 could be reinterpreted as a central (grassy) Type 9 circumscribed by Type 5s. However, often the social-functional characteristics emerging from crossing into a Type 6 are distinct even when there is an internal arrangement accommodating traversing by motorised transport. This is emphasised by Type 5s directing interaction onto a Type 6 (rather than alongside it as is more usual for Type 9s). Materially it is common to find architecturally elaborate Type 1s along a Type 6, making use of the wider perspective afforded by the open presentation of Type 6s.

**Figure 7.**
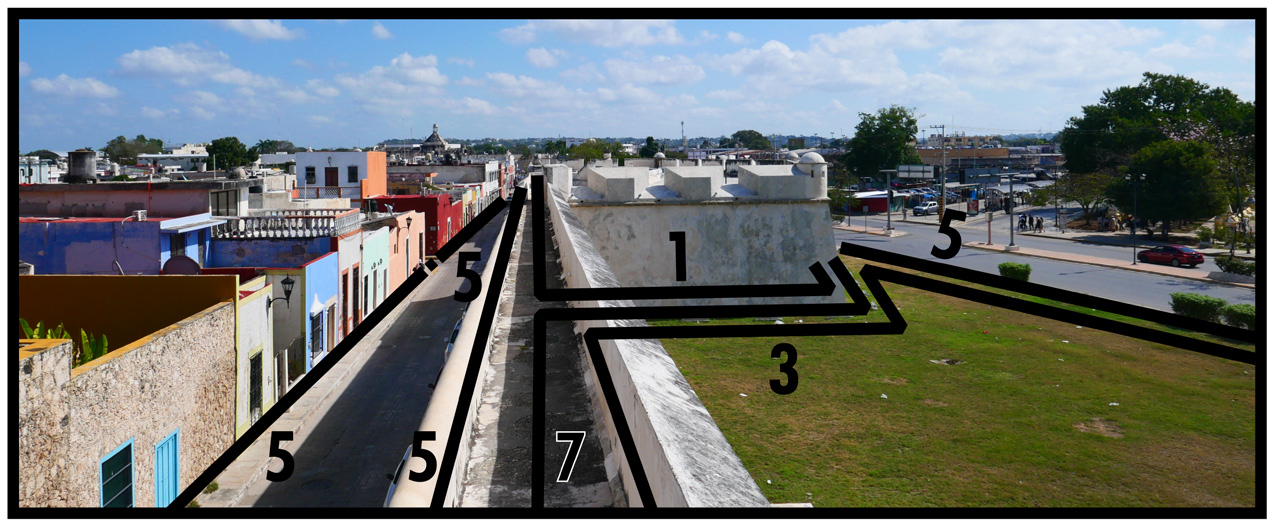
Boundary Line Type 7 in context.

Enclosing boundaries (Type 7) utilise the material property of stringently closable impermeability varying in scale, creating seclusions of elaborate, often large, and complex subsets of a BLT configuration which do not depend on each other socio-spatially beyond this material circumscription. When not exercising its closability, this subset could function as fully integrated into a larger built environment. Its material construction can impede open socio-spatial relations in everyday life, but will be especially restrictive from both outside and on the inside when closed. Just like other dominants, Type 7s create a materially emphasised power relation in socio-spatial functioning. Note that if a Type 7 only encompasses a subset of solid dominants and their associated configurative complexes, this implies the existence of a Type 8 interconnecting the subset; essentially a closable Type 8, cf. Type 3 above. As shown in Figure 7, common occurrences of Type 7s include city walls or other (smaller) fortified complexes, while gated communities are also common in some geographies. In Figure 7 the bastion is identified as a Type 1, becoming part of the Type 7 circumscription, while the field of fire is identified as a Type 3. The logic could be applied that the city wall itself is also a ‘bounded’ occupiable space. For present purposes, the material emphasis of the Type 7 definition already captures the specificity of the socio-spatial functioning of walling. Just like Type 1, a Type 7 requires at least one Type 2 in order to participate in the inhabited built environment.

**Figure 8.**
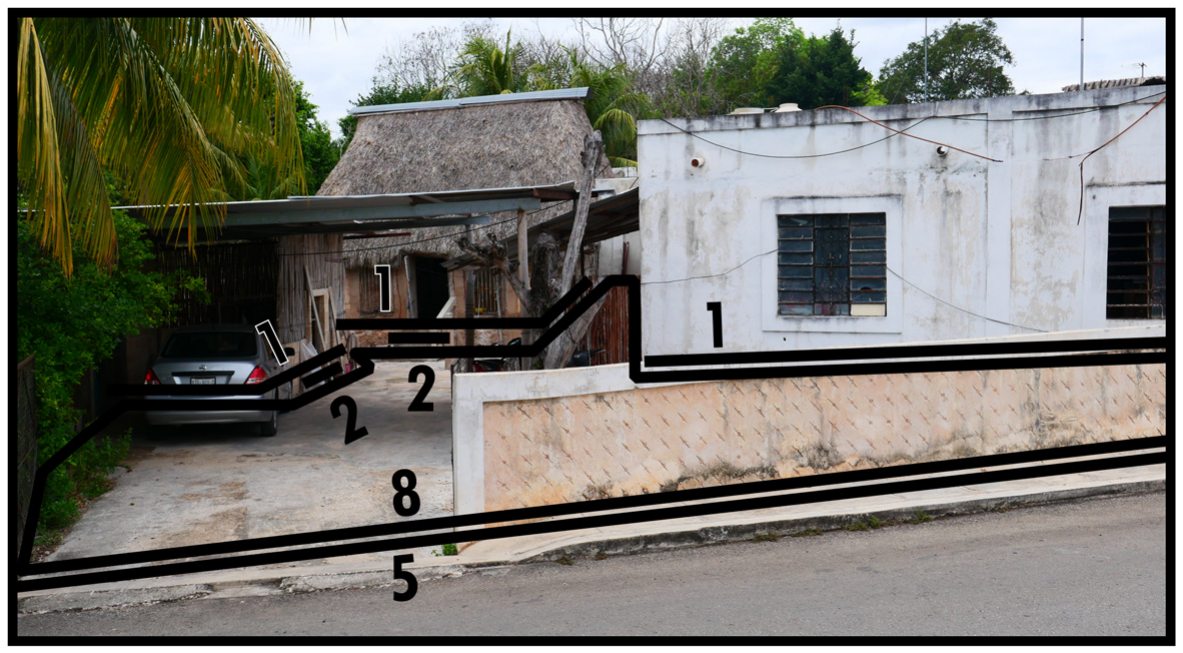
Boundary Line Type 8 in context.

Similar to Type 7, Type 8 also binds a subset of boundaries together. However, the principle of mutuality emerges from the simultaneous association with several Type 1s (and potentially related Type 3s). This creates an immediate relation between these Type 1s, forming a shared configurative complex defined from the inside without favouring orientation that is connected to outside thoroughfare (see Figure 8). In Figure 8 the Type 8 envelops the Type 1s. However, Type 8s could also be positioned so that they interconnect a subset of buildings only to the front or back; acting as a kind of amalgamating Type 3. If there is material evidence for impermeability circumscribing a mutually bounded configurative complex, the Type 8 arranging the internal relations gains a Type 7 that specifies outside relations. Most commonly occurrences of Type 8s refer to groups of buildings in a shared plot, which can happen in housing, such as in Figure 8, cul-de-sacs or shared gardens and facilities between a group of housing units. Type 8s can also be expected in architectural complexes with specialist commercial, civic, or industrial functions.

### Opening & neutral boundaries (9, 10)

**Figure 9.**
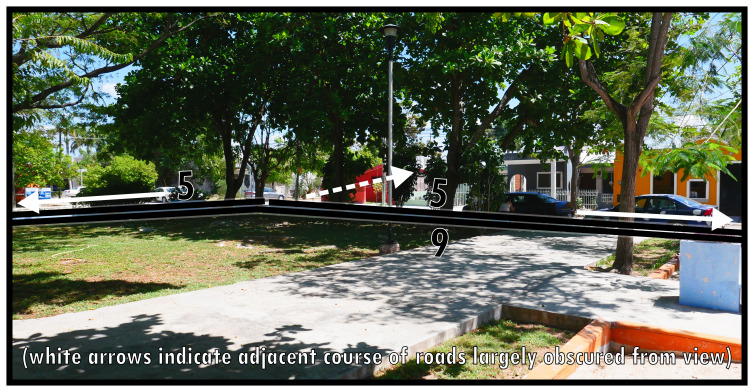
Boundary Line Type 9 in context (a multifunctional landscaped park).

**Figure 10.**
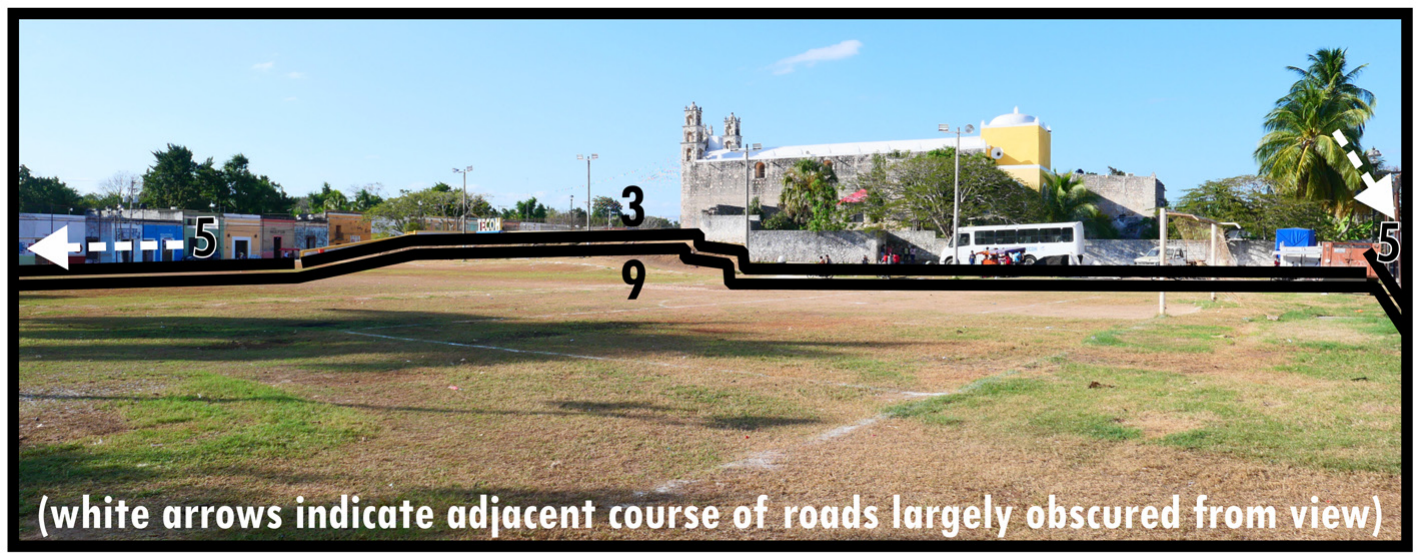
Boundary Line Type 9 in context (a sports field).

To illustrate the protean morphology and, often informal, social functioning of Type 9s, Figure 9 and Figure 10 provide two distinct examples: one an urban park, using an area along the roadside in a residential neighbourhood to create vegetation and shade for socialising, leisure, and play, the other conceived as a sports field reserved centrally in town being used informally with potential for an event space. Both instances also offer alternative thoroughfare or widen opportunities for mobility. Importantly, Type 9s are well-integrated into the surrounding configuration. Unlike Type 6 and Type 8 there are *only potential* relations to dominants (n.b. follies or maintenance structures could generally be considered as internal design features). The boundary tends to be broadly accessible and traversable, while thoroughfare is generally arranged alongside it rather than creating a focal access point (Type 8) or centring local mobility (Type 6). Gates and fencing may create some ambiguity about the closability of parks, which occasionally could render them dominants as Type 1s.

**Figure 11.**
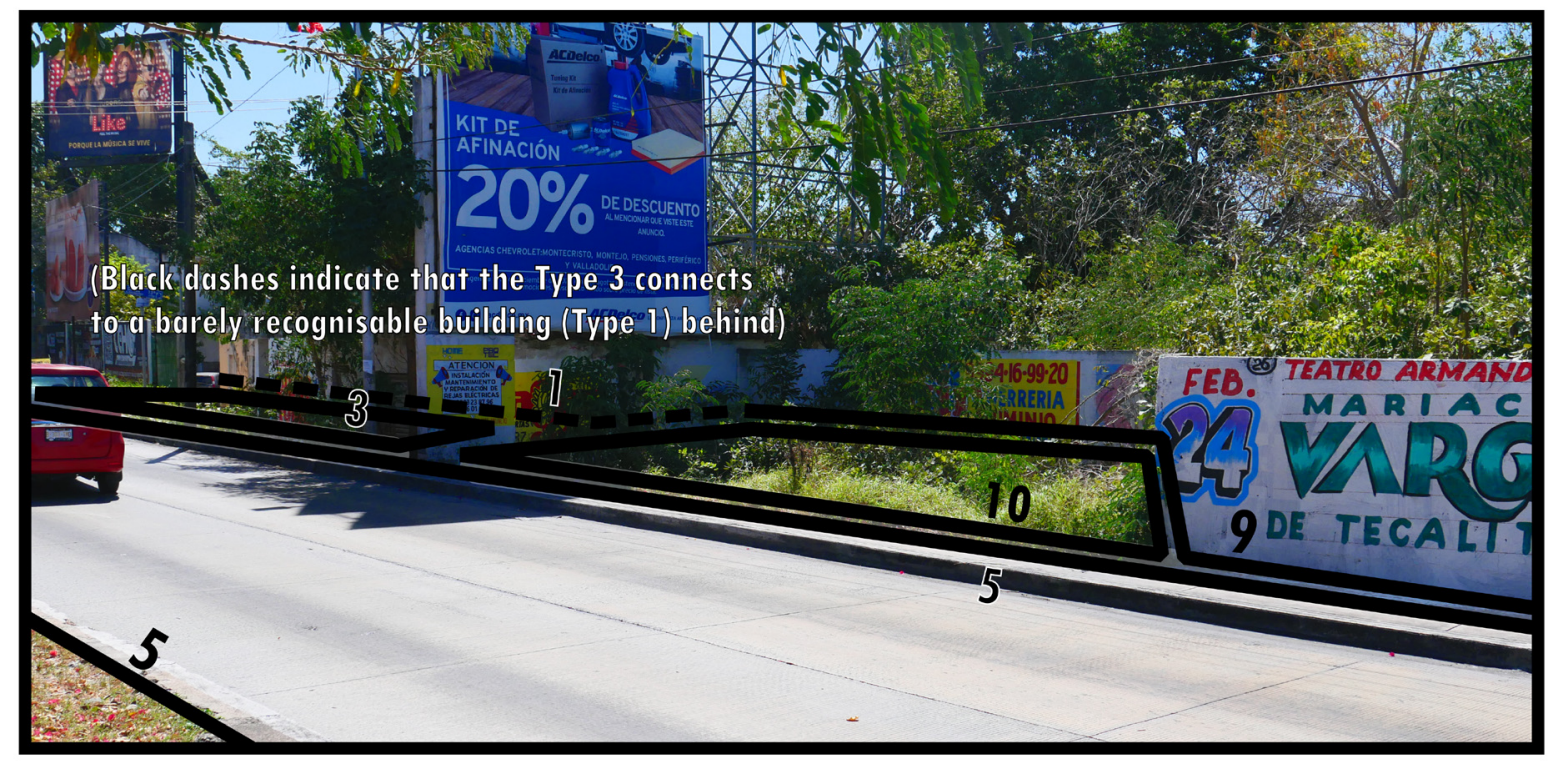
Boundary Line Type 10 in context.

Similarly diverse in form, if generally much less frequently occurring in high intensity urban developments, are neutral boundaries (Type 10). As shown in Figure 11, Type 10s pertain to instances of space which, essentially, are suboptimally used. While a materially easily perceptible distinction exists, this discrete spatial separation does not concur with a distinct residing socio-spatial system on all topological sides. Neutrality indicates that Type 10s are not internally defined, but circumscribe materially distinct spaces which remain after defining materially stronger boundaries along its outside (cf. physical holes as conceptualised by Smith and Varzi (2000), which are determined by surrounding elements and do not contain their own shape).

In Figure 11 the space described by Type 10 forms part of the roadside. While it could be assigned a distinct function, this area currently maintains the road layout due to an open connection. Crossing the open connection(s) of Type 10 boundaries does not significantly change socio-spatial interaction opportunities.

Type 10s often describe small areas that seem integrated, yet effectively they are left over after all surrounding space has become defined by social functions. This could be associated with small areas of soil, pavement, bedrock, or vegetation in intensely developed built environments, and in some instances with urban fallow. In Figure 11 we can appreciate the close relationship with urban fallow, as the Type 9 identified behind it describes a disused plot of land, but with undeniable material separation between the two.

### Negative definitions (11, 12, 13)

**Figure 12.**
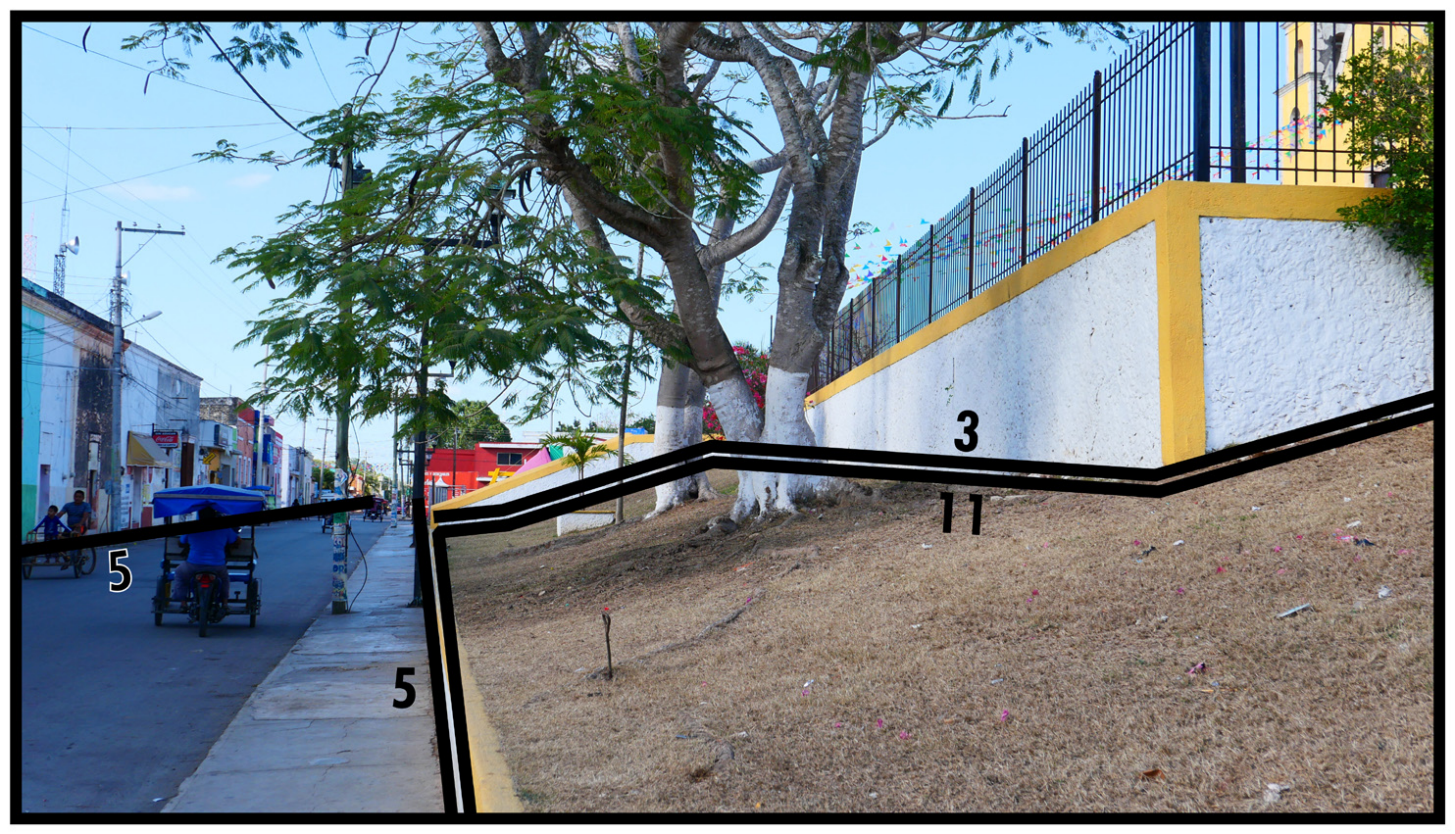
Boundary Line Type 11 in context.

**Figure 13.**
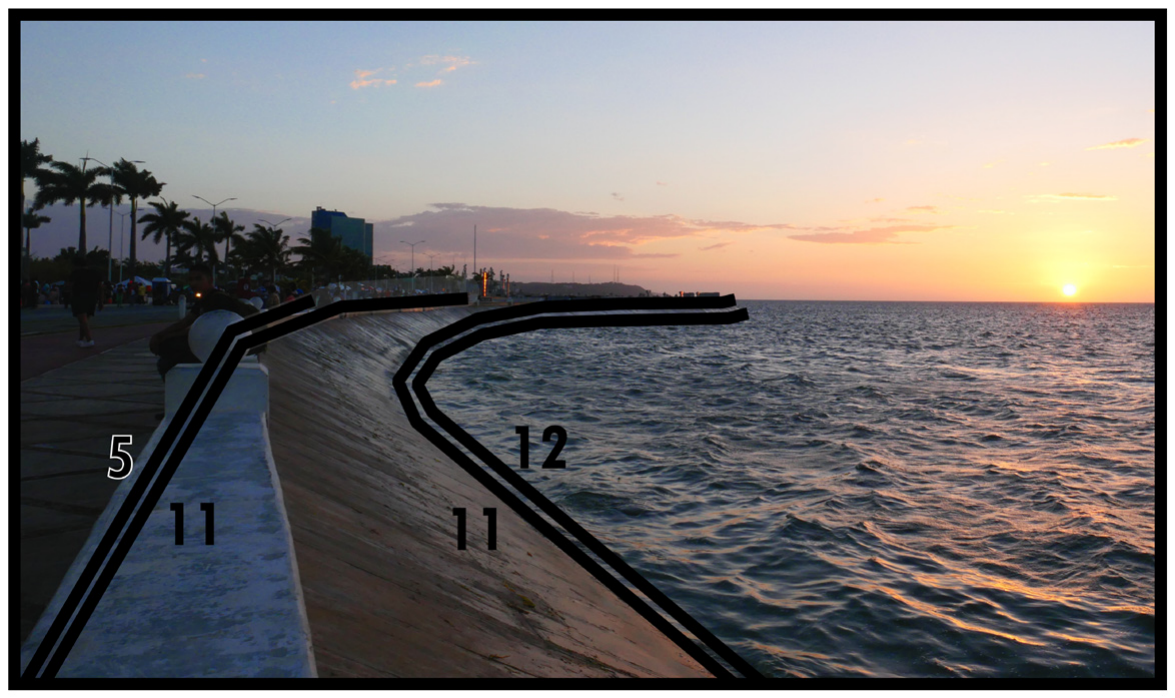
Boundary Line Type 12 in context.

Negativity concerning boundary line types refers to the phenomenon of areas of space which are not occupied by socio-spatial systems with a degree of permanence. When this occurs within a contiguous built environment it results from the physical impossibility for such occupation ( *i.e.* unoccupiability). The distinction between these two boundary line types refers to whether these spaces were constructed to be unoccupiable (man-made, Type 11) or unoccupiable due to physical characteristics that result from nonhuman processes (not man-made, Type 12). In Figure 12 and Figure 13 Type 11 refers to slopes resulting from past and present construction respectively. Electrical transformers, substations or water towers exemplify inaccessible structures. Canals and ponds obstruct access and often railways and motorways dissect built environments causing similar barriers to access, thus can be conceptualised as Type 11s (adaptable when technological transport systems are relevant to analyse). Since the Type 12 in Figure 13 represents the coastline, it could be confused with Type 13 (below). However, as a body of water cannot be occupied ^9^, Type 12 applies while also delimiting the built environment. More commonly Type 12s intersect or truncate the built environment as rivers, cliffs, or rocky outcrops (such as surface remains of schist in Manhattan, New York City).

**Figure 14.**
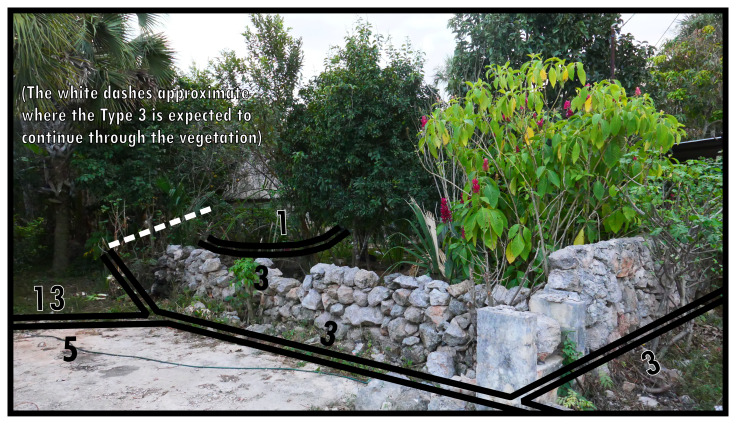
Boundary Line Type 13 in context.

Alternatively, negativity indicates that an environment commences which is predominantly formed by nonhuman processes, thus delimiting the extent of the contiguous built environment. This principle could also be used set an arbitrary area of study, thus delimiting the maximum extent of the boundary configuration under consideration. Boundaries that restrict the inhabited built environment in this way are identified as Type 13. In Figure 14, a dead-end street is where the contiguous built environment terminates. The palm leaf roof indicates the presence of a home hiding in the vegetation occupying much of the stonewalled plot.

## Reflections on applicability: built environments as spatial morphology composed of boundaries

Hall (1996: viii) wrote: ‘One could spend a lifetime on nothing but boundaries. This would be a worthwhile work’ when introducing Pellow’s (1996) anthropological volume on boundaries. My BLTs are first and foremost intended as a foundation for comparative social and cultural studies of built environments. As such it is capable of unburdening common mappings and (institutional) geographical data from predetermined, socio-culturally specific, use-space conflations. To that end it proposes a formulaic language or codification of how spatial morphology matters to social life at a rudimentary level. What results are emergent formal socio-spatially thick redescriptions of the spatial morphology that organises built environments. Even though the BLT definitions are fixed, albeit suspended in their practical adequacy ( *sensu* critical realism), for any given case the combinations and distributions in which they occur cannot be predicted. This means that while we can approach any given place (inhabited built environment) with BLTs, we may not necessarily find the same combinations of BLTs in each place and we certainly would expect them to differ in their frequency and distribution. This in turn permits particular kind of boundaries to play more dominant roles from one place to the next (see case demonstrations in Vis, 2018a). In Yaneva’s (2012: 4) words, BLTs help ‘to describe [and make explicit] the local treatments of the universal’.

Amin (2008: 12) posits that the ‘placement of time through materialization [...], repetition and rhythmic regularity [...], and juxtaposition [...] is its taming.’ As theoretical constructs, BLTs express a useful material reification of the both essential and commonplace constitutive role of boundaries in social life from which built environments develop. Precisely through their experiential and social constitutive theoretical foundations – the principles of differentiation and subdivision and the operation of seclusion – BLTs make a natural conceptual partner for long-term comparative studies on how built form develops. The materialisation of BLTs represents a state of establishment immediately followed by negotiation, modification, and potentially transformation as afforded by their properties (see Vis, 2013; Vis, 2018a). We appreciate ‘that boundaries may be both contested and consensual, depending on the perspectives and interests of the parties involved, [which] leads to a more dialectical understanding of their nature and transformations’ ( Rodman & Cooper, 1996: 94).

In other words, these boundary concepts provide the capacity for systematic social investigations of built environments as they develop, considering both the deep past of settlements and examples of vanished cultures. BLTs will help archaeologists to ‘engage conceptually with work in other disciplines to make effective ancient-modern comparisons, and […] to analyze (or reanalyze) our data so that we can address the topics and concepts of interest’ ( Smith & Peregrine, 2012: 15; see Vis, 2018a). Through a developmental lens, spatial morphology itself becomes a necessary constitutive part of social phenomena. Allowing the significance of the material presence of spatial morphology as a causal power in structuring social life and events avoids that social insights become largely detached from the space they originate in (cf. Zimmermann, 2012). Vice versa, conceptual ontologies of inhabited built environments arising from BLT applications will help to mitigate what Batty (2009: 194) describes as ‘considerable confusion about the way that the physical structure relates to human behaviour.’

In ensuring that BLTs’ theoretical foundations acknowledge that the materialisation of boundaries represent a time-space specific state, we understand that the repetition, rhythmic regularity, and juxtaposition of the occurrence of boundaries in the built environment do not ‘tame’ time. ‘Boundaries are not merely artifacts of the differentiation of places. They are intrinsically related to the actions they contain’ ( Rotenberg, 1996: 56). In fact, their causal powers will co-determine what will happen next. So, we might recognise BLTs as an ‘underlabourer’ for spatially conscious social research, akin Pratt’s (1995) description of critical realism as an ‘underlabourer’ for the research process.

As shown through the photographic illustrations, BLTs can be used simply as a transferable vehicle to think with and organise studies about events, activities, and land-use occurring in space or spatial development processes. Photographs offer an accessible approximation of lived experience. It is worth bearing in mind that ‘a photograph provides an instance of a phenomenon, not the essence of a phenomenon’ ( Hutson, 2012: 290). When annotating the BLTs we augment perceived reality by making the transferable essence of a material (social-spatial-physical) phenomenon explicit. The photographic illustrations demonstrate that through an individual’s perspective, (view and interaction), one’s experience and understanding of the nature of boundaries can remain limited. Inhabitants would get to know boundaries by moving through space and building up experience with negotiating and engaging with boundaries over time, and can be expected to interpret partial perception of boundaries according to their previous experience of similar situations.

In contrast, when BLTs are applied to maps, this affords us a totalising ‘god’s view’ ( Aitken & Craine, 2006; Monmonier, 1996; Morton *et al.* 2014; Vis, 2018b; Wood, 1992) through which BLTs act as a heuristic device to elucidate the significance of the material presence of spatial morphology to rudimentary social functioning. It is worth emphasising that their empirical identifiability following from their formal definitions permits the BLTs to be used in quantitative geographical analyses ( Vis, 2018a). However, since their definitions do rely on human and expert judgment their identification remains interpretive, as is, to some extent, judging which details from geographical input data to include or exclude. This reflects Galison (1998: 341), who emphasises: ‘On the side of the observer, there is a peculiarly human ability to seize patterns, and therefore to classify even when our algorithmic forms of reasoning fail. Subjectivity becomes an important feature of classification because the objects do not hold universal essential properties and because it is within our species’ nature to be able to classify univalently.’ While the BLTs themselves are argued to express essential and universal properties occurring within spatial morphology, their application currently relies on the judgments of Galison’s ‘empirically artistic’ human mind.

The proposed BLTs lay a foundation for future research and welcome triangulation or enrichment with other data, methods, and interpretations. The critical realist underpinning of the definitions should be regarded as an invitation to adapt and apply according to individual research objectives and data needs. Through applications and adaptations, the ontological redescriptions of built environments using BLTs should contribute a comparative base to themes that are socially, culturally, cognitively, emotively, or architecturally specific. Ultimately, approaching inhabited built environments through boundaries provides social scientific research with a spatial morphological canvas that is materially explicit.

## Ethics and consent

Ethical approval and consent were not required.

## Disclaimer

All photographs featured in this paper are taken by the author on locations across the states of Yucatan and Campeche in Mexico in the towns of Campeche, Halacho, Maní, Mérida, Tecoh, Tixkokob, and Umán.

## Data Availability

No data are associated with this article.

## Footnotes

^1^ Lynch’s (1984) discussion of conflation is insightful, but not instrumental in his work. He exemplifies a church, uniting a building and its meaningful functions as a religious centre and beacon to a community.

^2^ Walls are defined as extensions of the first architectonic datum of extracting and piling up material (mass) to create space division ( Van der Laan, 1983).

^3^ It is tempting to follow this analogy exclusively with geometric ordering of constructed material elements. For my purposes, all built features composing the built environment are not equated with Van der Laan’s (1983) specific concept of architectonic space. Materially speaking, boundaries frequently manifest without vertical form. Bear in mind that Van der Laan wrote from an architect’s perspective, proposing a design philosophy rather than analyses or formal redescription. Similarly, Van der Laan’s spatial intelligibility results from measuring its quantities.

^4^ It might seem as if this excludes the temporary settlements of nomadic societies. Yet, *e.g.* tents contain material mass and their arrangement generates spatial layouts following the same basic principles. For argumentative clarity this paper will not consider the particulars of ephemeral settling behaviours and the divergent affective properties of the materials employed. Nonetheless, fundamentally they can be considered in similar terms ( Delitz, unpublished work; Rapoport, 1978).

^5^ The discipline of archaeology is intrinsically bound to empirical material records. In the USA and Mexico archaeology forms an inherent part of the anthropological discipline, while elsewhere archaeology often finds itself classed as a humanities subject or liaised with historical disciplines, emphasising interpretive knowledge production. Some seek to (re)position archaeology as a social scientific discipline, asserting its pertinence to understanding human nature, societies, and development ( *e.g.* Smith *et al.*, 2012).

^6^ Varzi (2019) defines the term as “the theory of parthood relations: of the relations of part to whole and the relations of part to part within a whole”.

^7^ Exactly in which ways and to what degree the properties of material construction are permeable is difficult to determine without direct observation of physical affordances in terms of sensory functioning. Even a sturdy wall might permit aural communication, so which characteristics would completely remove sound travelling through walls (see also Rodman & Cooper, 1996)? Such assessment requires experiments and detailed documentation of material properties. In anthropology and (spatial) archaeology sensory approaches increase in popularity ( *e.g.* Boivin *et al.*, 2007; Bull & Back, 2003; Drobnick, 2006; Howes ed., 2005). I anticipate that boundaries could be enriched with such information.

^8^ Consistent information on the presence, location, and extent of entranceways is often absent in (mapped) geographical data. Pragmatically, any stretch of boundary with one or more entrances renders its material properties closable (rather than impermeable). Often this relationship can be surmised contextually. Where information on entranceways is available ( *e.g.* some architectural and archaeological plans) boundary line types can retain this information precisely. The material properties of impermeability and closability are ideally supported by direct evidence.

^9^ Unocupiability indicates that the surface cannot be inhabited or structurally utilised by a residing socio-spatial system. In certain instances, bodies of water are deliberately maintained to function as access ways ( *e.g.* Venice) or accommodate floating constructions ( *e.g.* The Netherlands). Steep slopes might be made tractable by stairs or funiculars. When physical evidence suggests a surface is rendered occupiable, other BLTs could apply.

## References

[ref-1] Abbott A : Things of boundaries. *Soc Res.* 1995;62(4):857–882. Reference Source

[ref-2] Aitken S Craine J : Guest editorial: affective geovisualizations. *Directions Magazine.* 2006. Reference Source

[ref-3] Amin A : Collective culture and urban public space. *City.* 2008;12(1):5–24. 10.1080/13604810801933495

[ref-4] Appleton J : The experience of landscape. London: John Wiley and Sons,1975. Reference Source

[ref-5] Arana López GN : La vivienda popular porfiriana: aportaciones a su estudio en Yucatán, Mérida. Editorial Dante, Mérida,2017. Reference Source

[ref-6] Batty M : Accessibility: in search of a unified theory. *Environ Plann B Plann Des.* 2009;36(2):191–194. 10.1068/b3602ed

[ref-7] Bell CM : Ritual: perspectives and dimensions. Revised Edition. Oxford: Oxford University Press,2009. Reference Source

[ref-8] Boivin N Brumm A Lewis H : Sensual, material, and technological understanding: exploring prehistoric soundscapes in South India. *J R Anthropol Inst (N.S.).* 2007;13(2):267–294. Reference Source

[ref-9] Bollnow OF : Lived-Space. *Philosophy Today.* 1961;5(1/4):31–39. 10.5840/philtoday1961513

[ref-10] Bourdieu P : Outline of a theory of practice. Cambridge: Cambridge University Press,1977. 10.1017/CBO9780511812507

[ref-11] Bull M Back L, eds. : The auditory culture reader. Oxford: Berg,2003. Reference Source

[ref-12] Conzen MRG : Alnwick, Northumberland: a study in town-plan analysis. *Institute of British Geographers Publication*. London: George Philip,1960;27. 10.1177/0309132509334948

[ref-13] Csordas TJ : Embodiment as a paradigm for anthropology. *Ethos.* 1990;18(1):5–47. Reference Source

[ref-14] Deleuze G Guattari F : A thousand plateaus: capitalism and schizophrenia. Minneapolis: University of Minnesota Press,1987. Reference Source

[ref-15] Delitz H : Gebaute gesellschaft: architektur als medium des sozialen. Frankfurt/New York: Campus Verlag,2009. Reference Source

[ref-16] Delitz H : Architecture as medium of the social: towards a sociological theory of built space. Trans. R. Gorny and K. Card. unpublished manuscript.

[ref-17] Dovey K Pafka E Ristic M : Mapping urbanities: morphologies, flows, possibilities. New York: Routledge,2017. Reference Source

[ref-18] Drobnick J, ed. : The smell culture reader. Oxford: Berg,2006. Reference Source

[ref-19] Fletcher RJ : Materiality, space, time, and outcome. In: *A Companion to Archaeological Theory*. ed. J.L. Bintliff, Oxford: Blackwell,2004;110–140. 10.1002/9780470998618.ch7

[ref-20] Galison P : Judgment against objectivity. In: *Picturing Science Producing Art*.eds. C.A. Jones and P. Galison, London: Routledge,1998;327–359. Reference Source

[ref-90] Gibson J : The ecological approach to visual perception. Houghton Mifflin, Boston.1979.

[ref-21] Hägerstrand T : Space, time and human conditions. *In: Dynamic Allocation of Urban Space*. eds. A. Karlqvist, L. Lundqvist and F. Snickars, Westmead: Saxon House,1975;3–14.

[ref-22] Hall ET : Foreword. In: *Setting Boundaries: The Anthropology of Spatial and Social Organisation*. ed. D. Pellow, Westport: Bergin & Garvey,1996;vii–viii. Reference Source

[ref-23] Harris R Smith ME : The history in urban studies: a comment. *J Urban Aff.* 2011;33(1):99–105. 10.1111/j.1467-9906.2010.00547.x

[ref-24] Hillier B : Space is the machine: a configurational theory of architecture. London: Space Syntax,2007(1996). Reference Source

[ref-25] Hillier B Hanson J : The social logic of space. Cambridge: Cambridge University Press,1984. 10.1017/CBO9780511597237

[ref-26] Hodder I : Entanglement: an archaeology of the relationships between humans and things.Malden: Wiley-Blackwell,2012. 10.1002/9781118241912

[ref-27] Howes D : Introduction.In: *Empire of the Senses: The Sensual Culture Reader.* ed. D. Howes, Oxford: Berg,2005;1–17.

[ref-28] Howes D : Empire of the senses: the sensual culture reader.Oxford: Berg,2005. Reference Source

[ref-29] Hutson S : Unavoidable imperfections: historical contexts for representing ruined maya buildings. In: *Past Presented: Archaeological Illustration and the Ancient Americas.* ed. J. Pillsbury, Washington: Dumbarton Oaks,2012;283–316. Reference Source

[ref-30] Ingold T : The perception of the environment.London: Routledge,2000. 10.4324/9780203466025

[ref-31] Ingold T : Bindings against boundaries: entanglements of life in an open world. *Environ Plan A.* 2008;40(8):1796–1810. 10.1068/a40156

[ref-32] Jessop B Brenner N Jones M : Theorizing sociospatial relations. *Environ Plan D.* 2008;26(3):389–401. 10.1068/d9107

[ref-33] Jones R : Categories, borders and boundaries. *Prog Hum Geogr.* 2009;33(2):174–189. 10.1177/0309132508089828

[ref-34] Kent S : Partitioning space: cross-cultural factors influencing domestic spatial segmentation. *Environ Behav.* 1991;23(4):438–473. 10.1177/0013916591234003

[ref-35] Koch A : Autopoietic spatial systems: the significance of actor network theory and systems theory for the development of a system theoretical approach of space. *Soc Geogr.* 2005;1(1):5–14. 10.5194/sg-1-5-2005

[ref-36] Kropf KS : Urban tissue and the character of towns. *Urban Des Int.* 1996;1(3):247–263. 10.1080/135753196351029

[ref-37] Kropf KS : Aspects of urban form. *Urb Morphol.* 2009;13(2):105–120. 10.51347/jum.v13i2.3949

[ref-39] Lamont M Molnár V : The study of boundaries in the social sciences. *Annu Rev Sociol.* 2002;28:167–195. 10.1146/annurev.soc.28.110601.141107

[ref-40] Latour B : Where are the missing masses? the sociology of a few mundane artifacts.In: *Shaping Technology-Building Society: Studies in Sociotechnical Change.* ed. W. Bijker and J. Law Cambridge, Mass.: MIT Press,1992;225–259. Reference Source

[ref-41] Lawrence RJ : The multidimensional nature of boundaries: social classifications, human ecology, and domesticity.In: *Setting Boundaries: The Anthropology of Spatial and Social Organization.* ed. D. Pellow, Westport: Bergin & Garvey,1996;9–36.

[ref-42] Lawrence DL Low SM : The built environment and spatial form. *Annu Rev Anthropol.* 1990;19(1):453–505. 10.1146/annurev.an.19.100190.002321

[ref-44] Löw M : The constitution of space: the structuration of spaces through the simultaneity of effect and perception. *Eur J Soc Theory.* 2008;11(1):25–49. 10.1177/1368431007085286

[ref-45] Lynch K : Good city form.Cambridge, Mass.: MIT Press,1984 (1981). Reference Source

[ref-46] MacEachren AM : How maps work: representation, visualization and design.New York: The Guilford Press,2004. Reference Source

[ref-47] Miller D : Material cultures: why some things matter.Chicago: University of Chicago Press,1998. Reference Source

[ref-48] Miller D : Materiality.Durham: Duke University Press,2005. Reference Source

[ref-49] Monmonier MS : How to lie with maps.Chicago: University of Chicago Press,1996. Reference Source

[ref-50] Morton SG Peuramaki-Brown MM Dawson PC : Peopling the past: interpreting models for pedestrian movement in ancient civic-ceremonial centres.In: S. Rau and E. Schönherr (ed.) *Mapping spatial relations, their Perceptions and Dynamics: The city today and in the past.* Heidelberg: Springer International,2014;25–44. 10.1007/978-3-319-00993-3_3

[ref-51] Pellow D : Setting boundaries: the anthropology of spatial and social organization. Westport: Bergin & Garvey,1996. Reference Source

[ref-52] Pratt AC : Putting critical realism to work: the practical implications for geographical research. *Prog Hum Geog.* 1995;19(1):61–74. 10.1177/030913259501900104

[ref-53] Pred A : The choreography of existence: comments on hägerstrand’s time-geography and its usefulness. *Economic Geography, Planning-Related Swedish Geographic Research.* 1977;53(2):207–221. 10.2307/142726

[ref-54] Pred A : Social reproduction and the time-geography of everyday life. *Geogr Ann Ser B.* 1981;63(1):5–22. 10.2307/490994

[ref-55] Pred A : Place as historically contingent process: structuration and the time-geography of becoming places. *Ann Assoc Am Geogr.* 1984;74(2):279–297. 10.1111/j.1467-8306.1984.tb01453.x

[ref-56] Pred A : Place, practice and structure: social and spatial transformation in Southern Sweden: 1750-1850. Cambridge: Polity Press,1986. Reference Source

[ref-57] Rapoport A : Nomadism as a man-environment system. *Environ Behav.* 1978;10(2):215–246. 10.1177/0013916578102005

[ref-58] Rapoport A : The meaning of the built environment: a nonverbal communication approach. Beverly Hills: Sage Publications,1982. Reference Source

[ref-59] Rodman M Cooper M : Boundaries of home in toronto housing cooperatives. In: *Setting Boundaries: The Anthropology of Spatial and Social Organization. *ed. D. Pellow, Westport: Bergin & Garvey,1996;91–110.

[ref-60] Rotenberg R : Tearing down the fences: public gardens and municipal power in nineteenth-century Vienna. In: *Setting Boundaries: The Anthropology of Spatial and Social Organization. *ed. D. Pellow, Westport: Bergin & Garvey,1996;55–70.

[ref-61] Sayer A : Abstraction: a realist interpretation. *Radical Philos.* 1981;28:6–15. Reference Source

[ref-62] Sayer A : Realism and social science. London: Sage,2000. Reference Source

[ref-63] Schütz A : The phenomenology of the social world. Evanston: Northwestern University Press,1967. Reference Source

[ref-64] Smith ME : Aztec city-state capitals. Gainesville: University of Florida Press,2008. Reference Source

[ref-66] Smith ME Feinman GM Drennan RD : Archaeology as a social science. *PNAS Early Edition.* 2012;109(20):7617–7621. 10.1073/pnas.1201714109 PMC3356624 22547811

[ref-65] Smith ME Peregrine P : Approaches to comparative analysis in archaeology. In: *The Comparative Archaeology of Complex Societies. *ed. M.E. Smith, New York: Cambridge University Press,2012;4–20. 10.1017/CBO9781139022712.004

[ref-67] Smith B Varzi AC : Fiat and bona fide boundaries. *Philos Phenomen Res.* 2000;60(2):401–420. 10.2307/2653492

[ref-68] Turner V : The ritual process: structure and anti-structure. New York: Aldine de Gruyter,1969. Reference Source

[ref-38] van der Laan DH : Architectonic space.Leiden: Brill,1983.

[ref-69] Varzi A : Mereology. *The Stanford Encyclopedia of Philosophy*(Winter 2012 Edition), ed. E. N. Zalta,2019. Reference Source

[ref-70] Vis BN : Built environments, constructed societies: inverting spatial analysis. Leiden: Sidestone Press,2009. Reference Source

[ref-71] Vis BN : Establishing boundaries: a conceptualisation for the comparative social study of built environment configurations. *paces Flows.* 2013;2(4):15–29. Reference Source

[ref-72] Vis BN : Boundary concepts for studying the built environment: a framework of socio-spatial reasoning for identifying and operationalising comparative analytical units in GIS. *In: Proceedings of Computer Applications and Quantitative Methods in Archaeology (CAA), Proceedings of the 40th International Conference, Southampton, UK,* 2014a. Reference Source

[ref-73] Vis BN : Mapping socio-spatial relations in the urban built environment through time: describing the socio- spatial significance of inhabiting urban form.In: S. Rau and E. Schönherr (ed.) *Mapping Spatial Relations, their Perceptions and Dynamics: The city today and in the past.*Lecture Notes in Geoinformation and Cartography, Heidelberg: Springer International:2014b;45–93. 10.1007/978-3-319-00993-3_4

[ref-74] Vis BN : The material logic of urban space. *Journal of Space Syntax.* 2016;6(2):271–274. Reference Source

[ref-75] Vis BN : Cities made of boundaries: mapping social life in urban form.London: UCL Press,2018a. Reference Source

[ref-76] Vis BN : Understanding by the lines we map: material boundaries and the social interpretation of archaeological built space.In: C. Siart, M. Forbriger, and O. Bubenzer (ed.) *Digital Geoarchaeology.*Natural Science in Archaeology. Cham: Springer,2018b;81–105. 10.1007/978-3-319-25316-9_6

[ref-77] Wallace S : Contradictions of archaeological theory: engagin critical realism and archaeological theory.Oxford: Routledge,2011. 10.4324/9780203843475

[ref-78] Wauchope R : Modern maya houses: a study of their archaeological significance.Carnegie Institution, Washington D.C,1938. Reference Source

[ref-79] Wood D : The power of maps.New York: The Guilford Press,1992. Reference Source

[ref-80] Woodward K Jones III JP : On the border with Deleuze and Guattari. In: *B/ordering Space.* eds. H. van Houtum, O.T. Kramsch, W. Zierhofer, Burlington: Ashgate,2005;235–248. 10.4324/9781315261782-26

[ref-81] Yaneva A : Mapping controversies in architecture.Farnham: Ashgate,2012. Reference Source

[ref-82] Yeung HW : Critical realism and realist research in human geography: a method or a philosophy in search of a method? *Prog Hum Geogr.* 1997;21(1):51–74. 10.1191/030913297668207944

[ref-83] Zimmermann K : Eigenlogik of cities: introduction to the themed section. *Urban Research & Practice.* 2012;5(3):299–302. 10.1080/17535069.2012.727544

