# Supplementary material for: Boundaries of the built environment: defining the significance of the material presence of spatial morphology in social life
Source: Open Res Eur. 2024 Jun 10;3:184. Originally published 2023 Oct 24. [Version 2] doi: 10.12688/openreseurope.16591.2 (PMC11549549; doi:10.12688/openreseurope.16591.2)
Supplement: Peer review history

# Reviewer report: Reviewer response for version 2

**DOI:** 10.21956/openreseurope.19066.r45642

**Published:** 2024-11-08

**Authors:** Oluwagbemiga Paul Agboola

**Affiliations:** 1 Istanbul Gelisim University,, Istanbul, Turkey

- The study presents a thoughtful and innovative approach to analysing inhabited built environments through the concept of "Boundary Line Types" (BLT), proposing a fresh ontological framework that emphasises socio-spatial and material boundaries over traditional, culturally specific architectural categories. The study succeeds in articulating the rationale behind introducing this new lens, grounded in a critical realist philosophy and multidisciplinary theories that consider the development and use of space. However, while the study is conceptually rich, it could benefit from clarifying several key aspects to enhance accessibility and depth:

- **Methodology**: the study introduces a novel set of "formally descriptive ontological concepts". These concepts should be elaborated in the methodology section. How do these differ from or improve upon existing methodologies? More specifically, providing examples of typical geographic or architectural categories that BLTs aim to supplement or replace would help underscore their practical significance. This would also make the study more accessible to readers less familiar with ontological distinctions in geographic and social science research.

- Furthermore, the term “ *Boundary Line Types*” is introduced as a core concept without much contextual background or preliminary definition, which might lead readers to question how BLTs are practically identified and applied within empirical research.

- Although the study mentions the potential for quantitative spatial analysis, it does not specify how BLTs might be operationalized or measured in a way that translates to empirical work. Examples of how BLTs have been applied or illustrative case studies could help readers understand the scope and limitations of the approach.

- The claim that BLTs enable " *diachronic and cross-cultural comparative social research*" is promising but could be more persuasive with a clearer explanation of how BLTs can transcend socio-cultural specificities inherent in traditional spatial categories. This point could strengthen the argument for BLTs as a universally applicable tool, rather than one constrained by specific socio-cultural or architectural contexts.

Is the argument persuasive and supported by appropriate evidence?

Yes

Is the work clearly and cogently presented?

Yes

Is the topic of the essay discussed accurately in the context of the current literature?

Yes

Does the essay contribute to the cultural, historical, social understanding of the field?

Yes

Reviewer Expertise:

Built environment; Environmental sustainability; Regenerative built environment

I confirm that I have read this submission and believe that I have an appropriate level of expertise to confirm that it is of an acceptable scientific standard.

## References

[1] : Built environment transformation in Nigeria: the effects of a regenerative framework. *Journal of Asian Architecture and Building Engineering* .2024;23(2) : 10.1080/13467581.2023.2238045 789-812 10.1080/13467581.2023.2238045

# Reviewer report: Reviewer response for version 2

**DOI:** 10.21956/openreseurope.19066.r41360

**Published:** 2024-06-27

**Authors:** Karl Kropf

**Affiliations:** 1 Oxford Brookes University, Oxford, England, UK

The replies from the author provide sufficient arguments for the position taken.  I have no further comments.

Is the argument persuasive and supported by appropriate evidence?

Partly

Is the work clearly and cogently presented?

Yes

Is the topic of the essay discussed accurately in the context of the current literature?

Yes

Does the essay contribute to the cultural, historical, social understanding of the field?

Yes

Reviewer Expertise:

Urban morphology, definitions, semiotics and language of built form, methods of analysis of built form, modes of description and inference in the built environment and applications in design

I confirm that I have read this submission and believe that I have an appropriate level of expertise to confirm that it is of an acceptable scientific standard.

# Reviewer report: Reviewer response for version 1

**DOI:** 10.21956/openreseurope.17908.r35876

**Published:** 2023-11-20

**Authors:** Karl Kropf

**Affiliations:** 1 Oxford Brookes University, Oxford, England, UK

The article provides a succinct presentation of a novel and innovative approach to the analysis of the built environment and the interweaving of social life with built form. The focus on boundaries has significant merit in allowing for engagement with both the material and ideational aspects of human-built form interrelations as well as a solid practical benefit when undertaking research where the data available is limited to ground level surveys.

 The overall structure of the article is clear and the language is equally clear and fluent.

 The introduction is well – and appropriately – referenced and situates the work in a diverse and nuanced philosophical and conceptual context that provides the analytical methods with robust support.

 The core section very usefully sets out both a summary and illustrated version of the Boundary Line Types with explanatory text that helps to clarify the definitions and openly and usefully identifies potential ambiguities.

 The closing section provides a thoughtful discussion of the potential, multiple applications of the methods and the benefits it could bring. In that discussion, the author points out the unavoidable subjectivity involved in classifying boundaries using this – or any other set of definitions.

 While accepting subjectivity is philosophically unavoidable, in my view, there are potentially ways in which the definitions could be constructed to enhance intersubjective concurrence. Such concurrence is clearly beneficial when seeking to encourage wider uptake of the method. The scope for interpretation should not be so wide that different researches come up with wildly different results when applying the same method to the same data.

 To that end, and in the spirit of the 'insistence on a consistent, coherent and comprehensive set of definitions', there are a number of questions that come to mind. Is there a way to make the definitions more tightly topological, which is to say, relational with respect to the range of different types. As with natural language, most _sets_ of definitions are essentially self-referential. We define words with other words. In order to make the definitions more consistent and coherent, is it possible to define them in terms of each other – elements from the same general category? This would avoid the potential issue of mixing attributes in the definitions and so reducing their consistency and coherence – and reliance on particular cases for clarification. There is a danger that the definitions still rely on more 'commonplace' attributes that are not fully acknowledge. While this is a step toward formalisation, it is not inconsistent with the aims of the method nor should it reduce the flexibility of the definitions to apply to the widest set of instances. On that point, the author beneficially notes the potential for ambiguities in application. In my view that point should be fully embraced. Ambiguities should not be viewed as aberrations to be eliminated but legitimate phenomena to be explored. Appropriately, one should seek to identify the boundaries of the ambiguities.

 Related to this point, the annotated photographs, in my view, do not always help to clarify the definitions. This is in part do to perspective convergence and overlapping and in part due to the method of annotation. What would be ideal is to include both a photographic perspective view and a plan view of the same site, both with annotations. In some cases, the lines drawn on the photo seem to follow a boundary that is hidden from view but there is no differentiation in the line to indicate that the boundary it is meant to signify is not visible. Also, the arrows showing 'road directions' are not entirely clear. This is where a plan view would be very helpful.

 Another thought is that it might be beneficial to illustrate the types within a single site so that the relationships between the types (as above) is more evident.

 In my view, the paper is very worthy of indexing subject to making improvements to the annotated photographs and, ideally, the inclusion of a corresponding plan representation. I would also encourage, either as part of this paper or subsequent papers to refine the definitions to be as 'self-referential' as possible in order to make a consistent set.

Is the argument persuasive and supported by appropriate evidence?

Partly

Is the work clearly and cogently presented?

Yes

Is the topic of the essay discussed accurately in the context of the current literature?

Yes

Does the essay contribute to the cultural, historical, social understanding of the field?

Yes

Reviewer Expertise:

Urban morphology, definitions, semiotics and language of built form, methods of analysis of built form, modes of description and inference in the built environment and applications in design

I confirm that I have read this submission and believe that I have an appropriate level of expertise to confirm that it is of an acceptable scientific standard, however I have significant reservations, as outlined above.

# Response

**Published:** 2024-04-11

**Authors:** Benjamin Vis

I thank Karl Kropf for his praise of my efforts and thorough consideration of the BLT definitions themselves and their presentation. I shall respond to his concerns point by point below. It is pertinent to point out that very different questions or concerns are raised here compared to the first peer review. No doubt, this reflects the highly interdisciplinary nature of the subject matter and the benefits that could be gained from researchers engaging with the ideas presented coming from very different disciplinary and methodological perspectives and with different requirements. However, I also take the divergent interests demonstrated by the peer reviewers as evidence that the current presentation effectively permits and invites this engagement. Therefore, revisions that would fundamentally change the framing of my arguments or the character of the presentation of the definitions are unwarranted without further substantive empirical work by me or others in the scientific community. In particular I note that the formulation of the definitions themselves were not directly critiqued in the first peer review report, which rather questioned their epistemological nature. I also draw attention to the fact that this article represents a reiteration of presenting an overview of the BLTs with a social scientific framing, using a different set of examples with enhanced observational discussions to illustrate them. This latter point is an indication of the consistency of definitions across publications, which is important to maintain when no concrete shortcomings are identified, and the stability within the applicability of the definitions across examples. Nonetheless, I recognise that this previous work was also authored by me, therefore empirical testing by others could further improve consistency and confirm validity and usability.   From this report some interrelated questions and suggestions arise, which I shall try to summarise into concrete points to respond to according to my interpretation of them.  

1. The first questions and concerns regard the potential for ‘enhancing intersubjective concurrence’, so that the chance of very divergent identifications of BLTs by multiple researchers in any given built environment is reduced, making results more consistent and trustworthy.

  The thought that other researchers would contribute to the possible refinement of the conceptualisation of boundary line types is heart-warming. Naturally, presenting the overview of BLTs in a social scientific context has the explicit aim of encouraging cross-disciplinary engagement with the definitions. However, it is important to note that the concern for intersubjective divergence is as yet unproven. While worries to this effect may be legitimate, it would only be when two or more researchers interpreting the same images or maps of built environments would indeed lead to significantly different results that fault could be sought with the definitions. I share the reviewer’s opinion that methodologically (maintaining comparability) relying on consistency between applications of the same concepts is fundamental. As discussed in the article, the definitions were conceived on the basis of critical realist epistemology, using iterative abstraction as a key exercise. In the literature cited, especially Vis 2018a, the detailing of this process and the sample data used is given more attention. While this cannot fully stand in for comprehensive intersubjective testing of the BLT definitions, it acts as the assurance for generally consistent applicability.  

1. The reviewer coins the possibility of making the definitions more ‘tightly topological’, which might be achieved by making them fully ‘self-referential’ or to define them in terms of each other. According to the reviewer further ‘formalisation’ should not affect the methodological objectives, nor the flexibility in application, or the valuable recognition of ambiguities. Ultimately, the reviewer sees this formalisation either as a task “of this paper or subsequent papers”.

  As stated above, recognising how boundaries (as causal powers of seclusion) manifest in built environment configurations through the process of iterative abstraction was the exercise through which distinct BLT definitions were produced. However, as the paper clearly explains, the recognition of the existence of different kinds of boundaries in built environments at all results from the constitutive phenomenological importance of ‘differentiation’ and ‘subdivision’. The operation of ‘seclusion’ (the way different connected spaces emerge) is essentially topological. In the second section I state: “Exactly because bounded spaces do not exist in isolation but occur in an environment (thus maintaining a relation or relations to the outside), it is recognised that the significance of the material presence of spatial morphology in structuring social life and subsequent place development consists of how seclusion operates as a causal power. […] The seclusion (causal power) each boundary effectuates is always relative to the context in which it occurs” In the third I underline that seclusion is the primary underlying principle on which boundaries operate: “Giving the operation of seclusion ontological primacy makes that the first boundary line type definition will determine the ability to close off (make impermeable) a space from undisruptive interaction from the outside, emphasising an internal assertion of seclusion.” Looking at these stipulations from the perspective of the reviewer’s concern, I note that the BLT definitions already benefit from topological tightness in that they all refer back to the primary existence of a boundary constituting the materially strongest form of ‘seclusion’, and that their ‘operation’ always departs from (or builds outward from) that first principle of seclusion. Each definition then specifies particular identifiable material and topological properties qualities. Some BLTs are also directly defined in terms of each other. Especially Type 2, Type 4 and Type 7, and to some extent Type 6, have other BLTs as prerequisites for their occurrence. Meanwhile the underlying assumption throughout is that BLTs only exist as an interconnected complex, the set together expressive of a particular perspective (or coherent frame of reference) on the built environment. Partially, I think that the expectation that further benefits could be gained from fully self-referential (or in terms of each other) definitions results from the same confusion caused by my incautious use of the term ‘ontology’ that the first peer review report alerted me to. Seeing the BLTs *as *an ontology comes with different requirements. Indeed, if the BLTs taken together would truly form an ontology, also all relationships between them should be defined. However, as I repeatedly state in the article, the relations between BLTs results from their application (identification) in empirical encounters with the built environment, which leads to BLT combinations. This terminological slippage has been addressed throughout the paper. Nonetheless, after a comprehensive identification effort, the occurrence of BLT combinations and how these relate does essentially form a conceptual ontology (a comprehensive redescription) of that built environment. While I appreciate that further formalisation need not be at odds with the overall aims I set out, there is the risk of defining (thus prescribing) more particular relations between BLTs. The more relations between BLTs are defined a priori, the more BLT combinations are predetermined. The net effect of this would be that, either, further distinct BLTs will become defined (i.e. more definitions than the contained in the current set), less of which can be expected to occur in a wide variety of built environments, or, that the same number of BLTs grow more specific in nature, in which case flexibility in application and comparison will be affected. Ultimately, I therefore agree, combined with point 1 above, that this consideration would be better placed as a result of (applied) engagement with the current set of definitions. It may emerge that the definitions could be tighter or indeed that more (or slightly revised) definitions are necessary or would prove more consistent. Currently, I am satisfied that a useful consistency in topological tightness and self-reference is achieved, but do not preclude the possibility that even greater consistency could be achieved in the future.  

1. The reviewer suggests that there is a “potential issue of mixing attributes in the definitions […] and reliance on particular cases for clarification. There is a danger that the definitions still rely on more 'commonplace' attributes that are not fully acknowledged.”

Since I personally do not use the term ‘attribute’ and the only particular cases used are the annotated photographs, I think this remark refers to the relatively generic ‘indicative examples’ listed in the Abridged BLT definitions table. I agree that in providing the service of referring to commonplace terms for easy recognition of spaces or situations in the built environment, there is a risk that someone applying BLTs subconsciously will refer more readily to their personal understanding of these commonplace terms than the more formally phrased empirical and social parts of the definitions. However, for general comprehension and also to clarify how the formulaic parts of the definitions can manifest as commonly recognisable built environment elements, I believe the benefits outweigh that risk. It would be down to the integrity of the researcher applying the terms to ensure that they do not slip into the habit of looking to identify ‘streets’ rather than ‘directing boundaries’.  

1. The reviewer argues that the photographs and annotations are not always sufficiently clear, due to boundaries partially obscured from view resulting from overlaps in the perspective, and road directions being insufficiently distinctive. A suggestion is to have annotated plan views accompany the photographs.

The perceived problems resulting from a photographic presentation of the BLTs are actually explicitly acknowledged and embraced in the article. I refer to my closing section: “When annotating the BLTs we augment perceived reality by making the transferable essence of a material (social-spatial-physical) phenomenon explicit. The photographic illustrations demonstrate that through an individual’s perspective, one’s experience and understanding of the nature of boundaries can remain limited. Inhabitants would get to know boundaries by moving through space and building up experience with negotiating and engaging with boundaries over time, and can be expected to interpret partial perception of boundaries according to the experience of similar situations.   In contrast, when BLTs are applied to maps, this affords us a totalising ‘god’s view’ (Aitken and Craine 2006; Wood 1992; Monmonier 1996; Morton *et al.* 2014, Vis 2018b) through which BLTs act as a heuristic device to elucidate the significance of the material presence of spatial morphology to rudimentary social functioning.” Because in previous publications (Vis 2014b, Vis 2018a) identifying BLTs from mapped data is elaborately discussed, the empirical research practice and assumptions of which are very different and quite specific in nature, it was a conscious decision to focus this present work on the more intuitively accessible (inhabitant’s) perspective from ‘lived experience’ that photographs afford as more closely connected to typical observations in the social sciences. Different from previous work, this article provides a richer discussion of the particularities of how BLTs manifest themselves in these photographs (notwithstanding reviewer 1’s alternative opinion on this), embracing any resulting ambiguities (as suggested in the peer review report). Besides this consideration of the research design for this article, unfortunately it is not possible to provide a plan view for each photograph. In few cases this is because the exact location of where photographs were taken is not known, but also because the most accurate mapping available to me would be the less than ideal Google Maps, which would need to be carefully prepared as compatible base data to apply BLTs to. In fact, I take the combined reviewers’ suggestion to heart that case-based photography next to plan view illustrations of BLTs as occurring in a single site accompanied by a very thick contextual consideration of their occurrence, might be a useful next research output. In line with my cited argument above, in my opinion there is a practical usefulness to presenting the BLTs in this photographic way, even if technically the exact site of the boundary is not always fully visible in the photograph. On the one hand side, this ‘perspective invisibility’ is mitigated by my annotations, while on the other it represents the ready recognisability and understanding of boundaries as they are encountered in lived experience (or, indeed, the urban surveyor, as the case may be). After considering the reviewer’s opinion on this (and acknowledging that reviewer 1 noted no problems with the photographs), is it a weakness or an asset in the context of social research that the exact site of some boundaries is (slightly) obscured from view? Especially as they are accompanied by detailed discussions, I am not sufficiently convinced that this is a problem. I also acknowledge, that just like intersubjective application, clarity of BLT recognition is likely to grow simply by increasing the variety of illustrations published. In this article a balance was sought between the space required to discuss each photographic illustration in some detail and the minimum number of photographs required to illustrate each BLT sufficiently. In addition, care was taken not to repeat socio-cultural contexts for which photographic illustrations have previously been published. I do agree that the arrows are not always as clear and consistent in terms of indicating the adjacent course of the roads. I stress here in response that recognising the presence of a road, generally concurring with the existence of a Type 5, is more important than knowing the direction in which it runs. Nonetheless, I have made revisions to the photographs concerned to clarify the presence of roads in relation to the main spatial subject of the photograph. I think these adjustments go some way to satisfy the concern highlighted by the reviewer. The visual distinction of these arrows, with regard to visibility, has also been further specified. Additionally, *all* photos have been revised to address the possible concern that the actual sites of boundaries are insufficiently visible or obscured from view. In the very few instances where a practically ‘invisible’ boundary had been annotated, this is now highlighted and explained within the image itself. The annotations have been adjusted to become slightly lighter (narrower), so that less of the scene is obscured by the lines. In some instances contrast is added to clarify the course of the boundaries annotated. I think these visual revisions have yielded subtle yet significant results in terms of preventing issues with the visibility and clarity of the boundaries exemplified in the images.    

1. “Another thought is that it might be beneficial to illustrate the types within a single site so that the relationships between the types (as above) is more evident.”

First, it is worth noting that in Vis 2014b and Vis 2018a the BLTs are presented as they occur on the mapping of a single site. Therefore, for the present article, there is no significant novelty value in repeating this exercise. Instead, the previous publications clearly acknowledge the value this way of presenting the BLTs has. Second, it must be recognised here that there is no necessity for all BLTs to occur in any single sample or site. At times, the potential occurrence of all types can be difficult to predict. In order to ensure this is properly understood, I have added explicit remarks to my argumentation that there is no requirement for any particular BLT (albeit the absence of some would be odd) to occur in any given built environment.

# Reviewer report: Reviewer response for version 1

**DOI:** 10.21956/openreseurope.17908.r35875

**Published:** 2023-11-13

**Authors:** Heike Delitz

**Affiliations:** 1 University of Regensburg, Regensburg, Germany

The article aims to provide a (rich) typology, differentiating thirteen “Boundary Line Types” (“BLT”), in order to give a base of comparisons between architectural cultures, or modes of institutions of “settled societies”. Also, the aim is to provide “a transferable vehicle to think with and organise studies about events, activities, and land-use occurring in space or spatial development processes”.

 The attention on the build environment is welcome, that is for architectural artefacts and their constitutive role for any social space, or the spatial structuring of society. Also, strongly welcome is an understanding of built environment as “carrying socio-culturally specific meaning”.

 Yet, the article also causes some questions or interventions:

 Why are the ‘BLT’ understood as “ontological” ones; and why is the aim an “ontology” (the meaning of ontology here should be explained and carefully introduced)? And what is to be understand under the title of a “critical realist basis” and in which sense is this fruitful here (in the question of the relation between architectural or infrastructural artefacts on the one side, and the social or society on the other side)? In which way do the author avoids a Eurocentric view, particularly when speaking of “development” of built space, and more urgently, when the BLTs are understood as “essential and universal”?

 Why is the aim to come to a “quantitative analysis”, or what are the potentials compared to qualitative ones? Here, and overall, the given short examples of the BLTs are too ‘formal’, too thin. It would be better to give only one or two examples, in a more extended way, providing a truly sociological, or social sciences’ analysis (showing in which way social inequalities, social differentiations, interactions or perceptions are architecturally instituted, within one or two ‘BL’ types, only) – for envisioned is an “understanding of how constructed environments facilitate social life”.

Is the argument persuasive and supported by appropriate evidence?

Partly

Is the work clearly and cogently presented?

Yes

Is the topic of the essay discussed accurately in the context of the current literature?

Yes

Does the essay contribute to the cultural, historical, social understanding of the field?

Yes

Reviewer Expertise:

Sociology of architecture, sociological theory

I confirm that I have read this submission and believe that I have an appropriate level of expertise to confirm that it is of an acceptable scientific standard, however I have significant reservations, as outlined above.

# Response

**Published:** 2024-04-11

**Authors:** Benjamin Vis

I thank Heike Delitz for engaging with my work and for her encouragement, while underlining the importance of the topics I address here. I also welcome the critical reflections on my writing that followed from the questions that she poses. I shall respond to her concerns point by point below. However, it is useful to point out that quite distinct questions and concerns are raised here when compared to the second peer review. No doubt, this reflects the highly interdisciplinary nature of the subject matter and the benefits that could be gained from researchers engaging with the ideas presented coming from very different disciplinary and methodological perspectives and with different requirements. However, I also take the divergent interests demonstrated by the peer reviewers as evidence that the current presentation effectively permits and invites this engagement. Therefore, revisions that would fundamentally change the framing of my arguments or the character of the presentation of the definitions are unwarranted without further substantive empirical work by me or others in the scientific community.   I note that most of the questions in this report are quite philosophical in nature, meaning that I shall provide a relatively detailed explanatory discussion in response. These discussions are based on and accompanied by direct quotations from the article, as I believe the nature and intentions of my arguments for the most part had been clearly set out in the original text. Highlighting these passages demonstrates this, however, I will note when I agree that my phrasing could lead to undue confusion, which I have addressed in my revision of the text.  

1. “Why are the ‘BLT’ understood as “ontological” ones; and why is the aim an “ontology” (the meaning of ontology here should be explained and carefully introduced)?”

I agree that understanding why the conceptualisations are ‘ontological’ is important, or, indeed, why to aim for an ‘ontology’ at all. As early as in the introduction I am explicit about in which sense the socio-spatial conceptualisations of boundaries is to be understood as ‘ontological’ (note that this is distinct from forming a full ‘ontology’). It is also useful to note that work according to critical realist philosophy of science has a natural proclivity to be preoccupied with ontologies and ontological reasoning. So, arguably, accepting this is a work largely realised within a critical realist frame of reference is already an explanation as to why ontological reasoning is a central concern. Through the lens of this question, I soon realised that both in my abstract and in the closing statements, my writing seems to have suffered some terminological slippage. My less careful use of the term ontology in these parts would unintentionally lead to the expectation that conceptualisations as definitions themselves form an ‘ontology’. The reason for this is that effectively an ontology of the built environment does emerge from the comprehensive application of all BLTs, which is why cursorily I often think of the set of boundary concepts as an ontology of BLTs. I should have been more careful with this. While in my explanatory and specifying argumentation it remains clear that is not the case, I have revised all instances of the term ‘ontology’ in the text and especially adjusted the formulated of phrases in the abstract and closing statement to address this confusion, as well as adjusting the subheading of section 3.1 to be clearer about what my arguments focus on. The following introductory passages address why and how the BLT definitions should be understood as ontological: “Here I will present an ontological set of socio-spatial conceptualisations of essential built environment elements. These concepts enable a redescription of the built environment based in fundamental, empirically observable, spatial-material information. The aim of accomplishing such rudimentary redescriptions is to advance the understanding of social life in constitutive relation to built environments, exclusively using as input data the spatial morphology (configuration, topology, composition, and shape) of the material environment that societies create for inhabitation. [...] instead of thinking of discrete spaces or spatial entities as a *fait accompli*, they emerge from the way they distinguish themselves to their outside, which is an ongoing process in social life. This directs our focus towards the constitution of boundaries and what their material manifestation consists of (Vis 2013).   The ontological ( *i.e.* comprehensively redescriptive) definitions of types of boundary ( *Boundary Line Types *(BLT)) I will present, can be identified empirically in the spatial morphology of the inhabited built environment (Vis 2014, 2018b).” Subsequently, the second section opens with the statement: “The ontological basis of the BLT definitions is formed by two rudimentary and linked principles of the human condition of inhabiting the world: differentiation and subdivision.” From these statements follows that the BLTs are ontological with regards to ‘social life in constitutive relations to built environments’ as applied to ‘the spatial morphology of the material environment that societies create for inhabitation’. As the second quote demonstrates, BLTs are ontological because they arise from an existential premise. Additionally, the boundary concepts are parts that, as they come to relate in application, will have comprehensively redescribed the phenomenon of the constitutive relation between social life and built environments (that is, the inhabited built environment). It also follows that because in the constitution of the inhabited built environment spaces emerge from distinctions, the concepts that capture these distinctions (thus, boundary concepts) are ontological and do not in and of themselves form an ontology, so that the rudimentary characteristics of boundaries allow differences to emerge within the idiosyncratic phenomenon of each unique place. This emergent character of ‘the inhabited built environment’ as a particular perspective is further explained in the third section. “[…] each boundary or even each line has two sides and therefore the definition of any boundary line type needs to be considered topologically and contextually from both sides. […] no outline that circumscribes a unit of built space can ever be defined by only a single boundary line type. A comprehensively defined boundary is contextually dependent on exactly what emerges from the spatial morphology present on both sides.” “We recognise that boundaries should always be considered from two sides and that there may be mereological relationships and topological specifications that result from their material and morphological properties. Therefore, it becomes an expectation that the significance of spatial morphology to the inhabitation of built environments is only revealed through combinations of BLTs operating along the same boundary. The fact that boundaries do not become defined by a single act of defining also stresses their emergent logic and complexity.” Further, I pay attention to the mereology of spatial morphology, which recognises the possibility of additional boundary specifications based on “subsuming or uniting a whole subset of boundaries”. I hope to have reminded the reviewer that the article thus carefully sets out exactly what is meant by my frequent references to the term ‘ontology’ in this context, but also to what use.

1. In which way do the author avoids a Eurocentric view, particularly when speaking of “development” of built space, and more urgently, when the BLTs are understood as “essential and universal”?

Continuing the previous explanation, exactly the ontological nature of the BLTs, where from their combination emerges the redescription of boundaries in the built environment, means that there is an opportunity for their application wherever we recognise that an ‘inhabited built environment’ exists. It is not predetermined in any way which BLTs occur, nor, largely, how they will combine. The empirical situation encountered determines the identification of BLTs and their combinations emerge from these identifications. I quote a pertinent turn of phrase already quoted above: “A comprehensively defined boundary is contextually dependent on exactly what emerges from the spatial morphology”. However, responding to this concern alerted me to the omission of making explicit within the article that there is no necessity for any particular BLT to occur within any built environment. Even though it can be logically expected that many of the same BLTs will occur in most built environments, there is no requirement for all BLTs to be identified in a single built environment, and certainly not within the same compositions or with the same intensity, or indeed the same combinations to describe the boundaries composing the spatial morphology. While this could be surmised from section 3.1 I have now made an adjustment to make this aspect explicit so no confusion can occur. I also refer to another paragraph explicitly explaining the balance of ‘universal application’, as in sufficiently general for wide ranging comparisons, and specificity resulting from the empirical data one is confronted with: “[…] comparative application along the lines of Kropf’s (2009, paraphrased) insistence on a consistent, coherent and comprehensive set of definitions, which are general enough to be used comparatively in all thinkable contexts, yet specific enough to be identified as analytical units in the empirical reality of datasets. When applied, the BLTs will still resemble the spatial entities that socio-culturally specific or commonplace categories in geographical data ( *e.g.* house, street, garden, *etc*. as indicated in the final column of Table 1) recognise, but these entities will have been redescribed.” So, taking that we can agree that inhabited built environments exists the world over, and accepting that comparative studies need not impede the appreciation of cultural differences among them, I am not sure that this concern has adequate foundation. Naturally, one could argue that the epistemology (comparative, critical realist) that this work represents is western or Anglo-American. It is also worthy to acknowledge, notwithstanding the European colonial presence in Mexico, that all illustration and most examples cited within this article are non-European. This was also an important consideration for me writing this article, as previous publications about the BLTs used illustrations from a variety of European contexts. Nonetheless, it is through the process of iterative abstraction through which the BLT definitions were conceived, as documented in frequently cited literature in this article (Vis 2014b, 2018a), that the effective application of the BLTs in a precolonial Maya city shows that the identifications of BLTs is capable of articulating cultural difference through applying ‘universal’ concepts. In addition, the term ‘development(al)’ should be considered within the context and terminology used throughout this work, such as emergence and constitution, or change over time. It seems to me that mentioning it in this question would introduce a new frame of reference within which ‘development’ could assume some kind of normativity, which is nothing to do with this work. Finally, the full phrase that is highlighted talks about BLTs expressing ‘essential and universal properties’. In response to possible confusion about what this means, I have adjusted the phrase slightly to indicate these are properties occurring in spatial morphology. This should avoid the sense that, for example, universal cultural values or meanings could be contained in the BLT definitions.  

1. And what is to be understand under the title of a “critical realist basis” and in which sense is this fruitful here (in the question of the relation between architectural or infrastructural artefacts on the one side, and the social or society on the other side)?

  I appreciate (as did Wallace 2011, referred in my work) that the use of critical realism is unusual in the thematic context of material evidence. With that in mind, a wider discussion on the fruitfulness and potential of critical realist theory and epistemology in built environment research could well be worthwhile, but I believe lies comfortably outside the scope of the current work. The exact phrase the reviewer refers to is a shorthand explanation used in the abstract, where I argue that the shorthand suffices: “[…] the definitions of the BLTs, which are formulated on a critical realist basis and rooted in a multidisciplinary body of theory concerning the development and inhabitation of built space.” Readers are not deprived from knowing what this ‘critical realist basis’ is understood to be. In fact, while there is use of critical realist thinking and terms throughout the article, two passages clearly express how critical realism has been instrumental.   “[…] the concrete foundation of their definitions (Vis 2018a) combines constitutive phenomenology (Schütz 1967) with Gibsonian (Gibson 1979) affordance to precisely articulate their material character using archaeological adaptations of critical realist philosophy (Wallace 2011). The socio-spatial causal powers of boundaries are recognised in topological relations to their environment through empirical iterative abstraction (Sayer 1981, 2000; Yeung 1997). Meanwhile, the emergent nature of boundaries’ material character in constantly developing environments made for inhabitation produce a perspective on the built environment that recalls Pred’s ‘transformation of nature’ and Bourdieu’s *habitus* (Pred 1977; Pred 1984, 1986; Vis 2018a; cf. Wallace 2011).” “[…] the numbering is generally representative of the iterative abstraction process (cf. Sayer 1981, 2000; Pratt 1995; Yeung 1997) through which they have been identified and do not express any further meaning. The BLTs have also been named in active voice to express their role in the inhabitation of the built environment. In line with iterative abstraction, their ontological conceptualisation is deemed practically adequate by having been tested through several instances of application (Vis 2014b, 2018a), but remains forever open to revision and refutation depending on empirical findings. Since the language used should avoid being socio-culturally prescriptive, the comprehensive definitions (first presented in Vis 2018a: 141–158) may come across formulaic, complex, and descriptive.” Briefly reiterating from these statements, critical realist ways of working and philosophical thinking in this work comprise the use of reasoning with ‘causal powers’, ‘conceptualisation through iterative abstraction’, ‘emergence’, ‘multidisciplinary triangulation’, ‘practical adequacy’, and the ‘fallibility of conceptual validity through empirical observation’. But, it is good to be reminded that critical realism in its basis is *ontological* in the sense of existential theory or as a way to approach or decide what is real (through ideas about cause, effect, and the validity of knowledge). Therefore, stating that the BLTs have resulted from critical realist reasoning also in itself explains the ontological concerns here.  

1. Why is the aim to come to a “quantitative analysis”, or what are the potentials compared to qualitative ones?

  I reviewed the places where I mentioned quantitative analysis. I do not indicate that my aim is to facilitate quantitative analysis. I point out that the BLTs (in mapped application) have the *potential* for quantitative analysis. This means there is a potential for qualitative (however rudimentary) concepts to immediately exist as a quantitative expression also. I remain convinced it is important to highlight this potential of the BLTs, while I otherwise recognise that the exact way or ways in which quantitative analyses could be realised is outside the purview of this article.  

1. Here, and overall, the given short examples of the BLTs are too ‘formal’, too thin. It would be better to give only one or two examples, in a more extended way, providing a truly sociological, or social sciences’ analysis (showing in which way social inequalities, social differentiations, interactions or perceptions are architecturally instituted, within one or two ‘BL’ types, only) – for envisioned is an “understanding of how constructed environments facilitate social life”.

  While I welcome the idea of future work focusing on specific types of boundaries as they become fully defined and described by BLTs, I disagree that the detail in the examples is lacking for the purpose of providing an overview for a broad social scientific audience. In a differently focused work, a scholar could easily decide to make the discussions of particular kinds of boundaries as thick as they want. I stress, however, that according to the logic I present in my article, this would most likely not be work focused on one or two BLT concepts in isolation, but the occurrence (incl. frequency, distribution, constructed variety, scale, etc.) of particular BLT combinations in one or more built environments. After all, the BLTs remain a relatively abstract and partial redescription of an empirical boundary. Only BLT combinations provide a full formal redescription. My explicit purpose from conception is to provide an overview. This overview would facilitate the kind of work the reviewer suggests as a possible next step.   I take exception to the implication that ‘true sociological or social sciences’ analyses must always include greater and deeper levels of contextual socio-cultural specifics. There is ample use for statistics and measurement in social research on the one extreme and for completely immersive socio-cultural (or socio-economic or socio-political) specificity at the other extreme. It is easily recognised that the BLT definitions, as they are, are quite far removed from statistics or measurements alone, while as a methodological and heuristic device perhaps equally far removed from socio-cultural specificity. This very fact permits comparative application without losing all qualitative significance. While there is no reason that the occurrence of a single BLT combination may be helpful in understanding a certain socio-spatial situation in a built environment, the ‘understanding’ referred to in the abstract (as cited by the reviewer) and in the article will thicken through comparative analyses. That is exactly what the BLTs are designed to do. At their ‘intermediary level’ of specificity, BLTs dramatically thicken the formal understanding of built environment elements as encountered or represented in maps which can support of social scientific analyses pursuing understandings amplified by considering greater contextual specificity.   I refer to the following passage of the closing section: “In ensuring that BLTs’ theoretical foundations acknowledge that the materialisation of boundaries represent a time-space specific state, we understand that the repetition, rhythmic regularity, and juxtaposition of the occurrence of boundaries in the built environment do not ‘tame’ time. ‘Boundaries are not merely artifacts of the differentiation of places. They are intrinsically related to the actions they contain’ (Rotenberg 1996: 56). In fact, their causal powers will co-determine what will happen next. So, we might recognise BLTs as an ‘underlabourer’ for spatially conscious social research, akin Pratt’s (1995) description of critical realism as an ‘underlabourer’ for the research process.”   And my closing sentence: “Ultimately, approaching inhabited built environments through boundaries provides social scientific research with a spatial morphological canvas that is materially explicit.”
